# Beyond Vasodilation in Pulmonary Arterial Hypertension: Integrating Remodeling-Directed Therapies into Risk-Guided Management

**DOI:** 10.3390/jcm15218621

**Published:** 2026-11-05

**Authors:** Ahmad Kharoufeh, Ahmad Omari, Bassam Awad, Mohamedanas Mohamedfaruk Patni, Shadha Nasser BaHutair, Haidy Alzaghal, Hammad Alqedrh, Ashfaq Ahmad Shah Bukhari, Hafiz Ahmad

**Affiliations:** 1 RAK College of Medical Sciences, Ras Al Khaimah Medical and Health Sciences University, Ras Al Khaimah 11172, United Arab Emirates; ahmad.21901070@rakmhsu.ac.ae (A.O.); bassam.21901019@rakmhsu.ac.ae (B.A.); haidy.24901036@rakmhsu.ac.ae (H.A.); hammad.25901035@rakmhsu.ac.ae (H.A.); 2 Department of Community Medicine, RAK College of Medical Sciences, Ras Al Khaimah Medical and Health Sciences University, Ras Al Khaimah 11172, United Arab Emirates; 3 Department of Obstetrics and Gynecology, RAK College of Medical Sciences, Ras Al Khaimah Medical and Health Sciences University, Ras Al Khaimah 11172, United Arab Emirates; shadha@rakmhsu.ac.ae; 4 Department of Physiology, RAK College of Medical Sciences, Ras Al Khaimah Medical and Health Sciences University, Ras Al Khaimah 11172, United Arab Emirates; ashfaq.ahmad@rakmhsu.ac.ae; 5 Department of Microbiology, RAK College of Medical Sciences, Ras Al Khaimah Medical and Health Sciences University, Ras Al Khaimah 11172, United Arab Emirates; hafiz@rakmhsu.ac.ae

**Keywords:** pulmonary arterial hypertension, pulmonary vascular remodeling, sotatercept, activin signaling, BMPR2, risk stratification, disease modification, combination therapy, precision medicine, right ventricular function

## Abstract

**Background/Objectives**: Pulmonary arterial hypertension (PAH) management is evolving beyond conventional vasoactive therapies toward strategies targeting pathways implicated in pulmonary vascular remodeling. Despite advances in combination therapy, substantial residual risk persists. The clinical introduction of sotatercept, targeting activin signaling, has expanded the therapeutic landscape. This narrative review critically evaluates the evidence supporting remodeling-directed treatment and examines how established recommendations, individualized therapeutic considerations, and investigational strategies can be distinguished within risk-guided PAH management. **Methods**: Clinical, translational, and mechanistic evidence was narratively synthesized, prioritizing randomized controlled trials, prospective investigations, major guidelines, and relevant mechanistic studies. Particular attention was given to differences in trial populations, endpoints, background treatment, safety, and evidence maturity. **Results**: Sotatercept has demonstrated incremental clinical benefits across functional, hemodynamic, and clinical-outcome measures in studied PAH populations, although trial heterogeneity limits indirect comparisons and long-term disease modification remains unproven. Other remodeling-directed approaches, including growth-factor inhibition, BMPR2 restoration, immune modulation, and metabolic targeting, remain investigational, with variable and frequently preliminary evidence. The proposed synthesis distinguishes guideline-supported therapeutic escalation from individualized clinical considerations and research hypotheses, emphasizing residual risk, treatment tolerability, and demonstrated incremental benefit. **Conclusions**: Remodeling-directed therapy expands the treatment options for PAH but does not replace established vasoactive treatment or risk-based escalation. Future priorities include defining optimal therapeutic sequencing, identifying responsive patient populations, and determining whether sustained clinical improvements translate into modification of disease progression.

## 1. Introduction

Pulmonary arterial hypertension (PAH) is a progressive pulmonary vascular disease characterized by precapillary pulmonary hypertension and elevated pulmonary vascular resistance (PVR), resulting in increased right ventricular (RV) afterload and potentially right heart failure and death [1]. Despite substantial therapeutic advances, PAH remains associated with considerable morbidity and residual mortality. Contemporary management therefore emphasizes early treatment, repeated multiparametric risk assessment, and timely therapeutic escalation when low-risk status is not achieved or maintained [1,2].

For several decades, targeted PAH treatment has primarily addressed three pathways involved in pulmonary vascular dysfunction: endothelin, nitric oxide–cyclic guanosine monophosphate, and prostacyclin. Pharmacological modulation of these pathways has improved clinical outcomes, and combination therapy is central to contemporary management. Parenteral prostacyclin therapy remains particularly important in severe or high-risk disease [1,2]. Nevertheless, many patients experience persistent symptoms, hemodynamic abnormalities, or clinical deterioration despite multidrug treatment, highlighting the need for additional therapeutic approaches.

Persistent disease activity reflects the complex pathobiology of PAH, which extends beyond vasoconstriction. Pulmonary vascular remodeling involves abnormal vascular cell proliferation and survival, endothelial dysfunction, inflammation, metabolic reprogramming, extracellular matrix alterations, and dysregulated growth-factor signaling [3]. These interacting mechanisms contribute to progressive vascular narrowing and obliteration, increased PVR, and RV pressure overload. Accordingly, therapeutic development has increasingly expanded toward pathways implicated in the initiation and maintenance of pulmonary vascular remodeling.

A major advance has been the clinical targeting of dysregulated bone morphogenetic protein (BMP)/activin signaling. In the phase III STELLAR trial, sotatercept, an activin signaling inhibitor administered alongside stable background therapy, improved exercise capacity and several secondary measures of disease severity [4]. The treatment framework developed following the 7th World Symposium on Pulmonary Hypertension recognized BMP/activin signaling alongside endothelin, nitric oxide, and prostacyclin as a therapeutically actionable pathway [2]. This development represents an expansion of therapeutic mechanisms beyond conventional modulation of vascular tone, although clinical efficacy does not independently establish histological reversal of pulmonary vascular remodeling.

Subsequent trials have broadened the evidence supporting activin signaling inhibition across different PAH populations. In ZENITH, sotatercept reduced the risk of a composite clinical endpoint involving death, lung transplantation, or hospitalization for worsening PAH among patients with advanced disease receiving background therapy [5]. However, differences in trial populations, baseline risk, background treatment, endpoints, and follow-up duration limit direct comparisons between studies. Moreover, the relative contribution of individual components to composite outcomes must be considered when interpreting treatment effects.

These developments raise important questions regarding therapeutic sequencing, patient selection, treatment burden, and the interpretation of clinical improvement. Although remodeling-directed therapies may provide benefits beyond conventional vasoactive mechanisms, improved exercise capacity, hemodynamics, biomarkers, or clinical outcomes do not necessarily establish durable reversal of pulmonary vascular pathology. Demonstrating disease modification requires evidence that treatment meaningfully alters the long-term disease trajectory, supported by sustained clinical outcomes and, where feasible, complementary structural or biological measurements [6].

Existing guidelines and consensus documents provide essential recommendations for risk assessment and therapeutic escalation. However, important uncertainties remain concerning how remodeling-directed therapies should be incorporated into treatment decisions when patients exhibit persistent intermediate risk, discordant risk indicators, substantial comorbidities, or limitations in treatment tolerability. Equally important is distinguishing clinically established treatment options from investigational strategies supported primarily by preliminary or mechanistic evidence.

Accordingly, this narrative review critically examines the biological rationale, clinical evidence, safety considerations, and unresolved questions surrounding remodeling-directed therapies in PAH. Its principal contribution is an evidence-calibrated synthesis that distinguishes guideline-supported management from individualized clinical considerations and investigational therapeutic hypotheses. Particular emphasis is placed on differences in evidence maturity, the interpretation of clinical trial outcomes, and the distinction between symptomatic improvement, sustained disease control, and potential disease modification. Rather than proposing an independently validated treatment algorithm, the review identifies how residual risk, treatment tolerability, mechanistic complementarity, and demonstrated clinical benefit may inform future therapeutic integration and research priorities.

## 2. Literature Search Strategy

A structured, topic-focused literature search was undertaken to identify clinical, translational, and mechanistic evidence relevant to pulmonary vascular remodeling, remodeling-directed therapies, and risk-guided management in pulmonary arterial hypertension (PAH). PubMed and Scopus were the principal databases, with emphasis on English-language publications from January 2020 through September 2026. Earlier landmark studies and major international guidelines were considered where necessary to provide historical and mechanistic context.

The search covered pulmonary arterial hypertension, pulmonary vascular remodeling, risk stratification, disease modification, combination therapy, endothelin signaling, nitric oxide–cyclic guanosine monophosphate signaling, prostacyclin signaling, BMP/activin signaling, BMPR2, sotatercept, right ventricular remodeling, growth-factor inhibition, inflammation, immune modulation, metabolic dysfunction, extracellular matrix remodeling, mechanotransduction, and precision medicine. Trial-specific searches addressed major studies relevant to established and emerging therapies, including AMBITION, TRITON, PULSAR, SPECTRA, STELLAR, ZENITH, HYPERION, IMPRES, TORREY, and PROSERA. Reference lists of relevant guidelines, reviews, and primary studies were also examined to identify additional publications.

Evidence selection prioritized randomized controlled trials, prospective clinical studies, long-term follow-up investigations, major international guidelines and consensus statements, and relevant mechanistic or translational research. Primary trial publications were preferentially used to support claims regarding efficacy, safety, hemodynamics, and clinical outcomes. Studies focused primarily on pulmonary hypertension groups other than Group 1 PAH were generally excluded unless they provided directly relevant mechanistic insights. Case reports, small case series, and studies with limited relevance to the review objectives were not emphasized.

Evidence was critically interpreted according to study design, population characteristics, prespecified endpoints, statistical significance, multiplicity control, follow-up duration, clinical relevance, and publication status. Particular attention was given to distinguishing confirmatory randomized findings from exploratory subgroup analyses, surrogate endpoints, and preliminary or sponsor-reported results. When evidence was conflicting, greater interpretative weight was assigned to adequately designed randomized trials and their prespecified primary outcomes. Negative primary endpoints were not reinterpreted as positive on the basis of nominally significant secondary or subgroup findings.

This review was designed as a narrative synthesis rather than a systematic review or meta-analysis. It did not employ a prospectively registered systematic-review protocol, formal quantitative pooling, or a systematic risk-of-bias assessment. Consequently, the synthesis should not be interpreted as an exhaustive inventory of all available evidence, and its conclusions remain subject to the selection and interpretative limitations inherent to narrative reviews.

## 3. Biological Basis for Persistent Disease and Therapeutic Resistance in PAH

Despite advances in combination therapy, pulmonary arterial hypertension (PAH) remains characterized by persistent vascular pathology and substantial residual clinical risk. Although improvements in pulmonary hemodynamics, exercise capacity, and symptoms indicate therapeutic benefit, these responses do not necessarily establish resolution of the underlying pulmonary vasculopathy. PAH involves interconnected abnormalities in endothelial function, vascular cell proliferation and survival, inflammatory signaling, cellular metabolism, extracellular matrix organization, and genetic regulatory pathways [3]. Understanding these mechanisms is essential for evaluating the rationale and limitations of therapies directed toward pulmonary vascular remodeling.

### 3.1. A Proliferative and Apoptosis-Resistant Vascular Phenotype

Pulmonary arterial endothelial cells, smooth muscle cells, fibroblasts, pericytes, and recruited inflammatory cells contribute to structural remodeling through altered proliferation, migration, survival, and intercellular communication [3]. These processes promote medial hypertrophy, intimal thickening, adventitial expansion, and, in advanced disease, complex obstructive vascular lesions. Progressive reduction in the functional pulmonary vascular cross-sectional area increases pulmonary vascular resistance (PVR) and right ventricular (RV) afterload.

Endothelial injury and abnormal repair may contribute to the emergence of cell populations exhibiting enhanced proliferation and resistance to apoptosis. Experimental investigations and analyses of human PAH tissue have implicated endothelial Krüppel-like factor 6 (KLF6) in angiogenic signaling, reduced apoptotic susceptibility, and stimulation of smooth muscle cell proliferation [7]. These findings illustrate how maladaptive vascular repair may contribute to persistent remodeling.

Mechanical and proliferative signaling can also reinforce one another. Experimental studies implicating tuberous sclerosis complex 2 (TSC2)/mechanistic target of rapamycin (mTOR) signaling suggest that changes in extracellular mechanical forces may sustain vascular cell growth and survival [8]. However, these observations remain primarily mechanistic and do not establish that targeting these pathways improves clinical outcomes in PAH.

### 3.2. BMPR2 Dysfunction and Imbalance Within the TGF-β Superfamily

Dysregulation of bone morphogenetic protein receptor type 2 (BMPR2) signaling is among the most extensively characterized molecular abnormalities in PAH. Pathogenic BMPR2 variants represent the most common established genetic cause of heritable PAH, while abnormalities in related signaling components further support the importance of this pathway in pulmonary vascular homeostasis [9].

Under physiological conditions, BMP signaling contributes to endothelial integrity and regulation of vascular cell growth, differentiation, and survival. Impaired BMPR2 signaling can favor endothelial dysfunction, abnormal proliferation, and maladaptive vascular remodeling. These abnormalities occur within the broader transforming growth factor-β (TGF-β) superfamily, where reduced protective BMP signaling may coexist with relatively increased activin- and TGF-β-associated activity [10].

At the intracellular level, this imbalance is frequently conceptualized as diminished BMP-associated SMAD1/5/8 signaling relative to activin/TGF-β-associated SMAD2/3 signaling. Although this model simplifies a complex and context-dependent signaling network, it provides an important biological rationale for interventions targeting activin-related signaling [10].

Sotatercept represents the principal clinical translation of this therapeutic concept. By acting as a ligand trap for selected TGF-β superfamily ligands, it modulates signaling associated with vascular growth regulation. Importantly, sotatercept should not be described as directly restoring BMPR2 receptor function, and its demonstrated clinical benefits should not be equated with confirmed histological reversal of pulmonary vascular remodeling.

### 3.3. Inflammation and Immune–Vascular Crosstalk

Inflammatory and immune abnormalities contribute to the complex pulmonary vascular environment in PAH. Perivascular immune cell accumulation, altered cytokine and chemokine signaling, and interactions between inflammatory cells and resident vascular cells may promote endothelial dysfunction, vascular proliferation, and extracellular matrix deposition [3].

Inflammatory signaling is closely interconnected with other remodeling mechanisms. Changes in extracellular matrix composition and stiffness can influence immune cell activity and vascular cell responses, while inflammatory mediators may further modify matrix production and proliferative signaling [11,12]. Interactions with BMPR2 dysfunction, oxidative stress, and metabolic abnormalities may contribute to persistence of the remodeled vascular phenotype.

However, inflammatory involvement varies across PAH etiologies and individual patients. Observational associations between inflammatory biomarkers and disease severity do not necessarily establish that suppressing a particular immune pathway will improve clinical outcomes.

Consequently, immune-directed treatment remains an investigational approach outside established indications for treating associated systemic inflammatory or autoimmune conditions. Future development will require identification of biologically relevant patient subgroups and demonstration of clinically meaningful benefit in adequately controlled studies.

### 3.4. Metabolic and Mitochondrial Reprogramming

Pulmonary vascular remodeling is accompanied by alterations in cellular energy metabolism that may support abnormal proliferation and survival. Experimental and translational studies have implicated changes in mitochondrial function, glucose utilization, fatty-acid metabolism, oxidative stress, and cellular biosynthetic pathways [3].

Human investigations have identified abnormalities in mitochondrial function and fatty-acid metabolism in Group 1 PAH, supporting the clinical relevance of metabolic disturbances beyond experimental models [13]. Nevertheless, the extent to which individual metabolic abnormalities are causal drivers, compensatory responses, or consequences of advanced disease remains incompletely defined.

Metabolic signaling also interacts with the extracellular mechanical environment. Increased matrix stiffness can activate yes-associated protein/transcriptional coactivator with PDZ-binding motif (YAP/TAZ)-dependent pathways, promoting metabolic adaptations such as glutaminolysis that may support vascular cell proliferation [11]. Related experimental work has implicated glutamine and serine metabolism in fibroblast collagen biosynthesis and vascular stiffening [12].

These observations support investigation of metabolic interventions, potentially in biologically selected populations. However, mechanistic plausibility and favorable experimental findings have not yet translated into sufficiently robust clinical evidence to establish metabolic targeting as routine PAH treatment.

### 3.5. Extracellular Matrix Remodeling and Vascular Stiffness

Extracellular matrix remodeling contributes both to the structural manifestations and biological progression of PAH. Increased collagen deposition and cross-linking, altered elastin organization, abnormal matrix turnover, and vascular stiffening modify the mechanical environment experienced by pulmonary vascular cells [3,11].

Mechanotransduction allows vascular cells to detect and respond to these mechanical changes. Experimental evidence indicates that matrix stiffness can activate YAP/TAZ-dependent transcriptional programs and interact with TSC2/mTOR-associated growth signaling, potentially promoting proliferation, metabolic adaptation, and further matrix production [8,11].

These mechanisms may establish reciprocal interactions between vascular stiffening and cellular remodeling. Increased matrix deposition alters mechanical forces, while mechanically activated cells may produce additional extracellular matrix components. Metabolic pathways involved in collagen biosynthesis can further reinforce this relationship [12].

Although these findings identify potential therapeutic targets, clinical interventions specifically directed at matrix remodeling or mechanotransduction remain at an early stage of development. Evidence supporting these approaches is predominantly preclinical, and their efficacy, safety, and optimal patient populations have not been established.

### 3.6. The Right Ventricle as a Determinant of Clinical Progression

The clinical consequences of pulmonary vascular remodeling depend not only on the severity of vascular obstruction but also on the capacity of the right ventricle to adapt to increased afterload. Early compensatory responses may include increased contractility and hypertrophy, helping preserve cardiac output and RV–pulmonary arterial coupling. Persistent pressure overload can ultimately lead to RV dilation, impaired contractile function, systemic venous congestion, and right-sided heart failure [14].

RV maladaptation involves complex changes in myocardial metabolism, fibrosis, inflammation, ischemia, mitochondrial function, and coronary perfusion [14]. Importantly, patients with similar pulmonary vascular hemodynamics may exhibit different degrees of RV adaptation and clinical risk.

Established PAH therapies generally improve RV loading conditions through effects on the pulmonary circulation. Although favorable RV functional changes may accompany treatment, validated pharmacological strategies specifically targeting intrinsic RV maladaptation remain lacking.

This distinction has implications for future therapeutic development. Demonstrating improvements in pulmonary vascular resistance alone may not adequately characterize treatment effects on RV recovery or long-term clinical outcomes. Complementary assessment using RV imaging, biomarkers, exercise capacity, and clinical events may provide a more comprehensive evaluation of therapeutic benefit.

### 3.7. From Pathobiology to Therapeutic Opportunity

The biological mechanisms described above provide a rationale for therapeutic approaches extending beyond conventional vasoactive treatment. However, their clinical readiness differs substantially.

BMP/activin signaling modulation has progressed to an established therapeutic option through randomized clinical trials of sotatercept. In contrast, approaches targeting growth-factor signaling, immune dysregulation, metabolic abnormalities, extracellular matrix remodeling, mechanotransduction, and intrinsic RV maladaptation remain investigational, with evidence ranging from preclinical observations to early-phase or selected later-phase clinical studies.

This distinction is essential when interpreting the emerging therapeutic landscape. Identification of a biologically relevant pathway does not establish that pharmacological intervention will produce meaningful clinical benefit. Similarly, improvements in hemodynamics, functional capacity, or biomarkers do not independently demonstrate durable alteration of pulmonary vascular pathology.

The principal therapeutic challenge is therefore to determine which biological mechanisms can be targeted safely and effectively, which patient populations are most likely to benefit, and whether treatment produces sustained improvement beyond established therapy. These questions require evaluation of clinical outcomes, treatment burden, safety, and durability rather than reliance on mechanistic plausibility alone.

Figure 1 summarizes the relationship between pulmonary vascular pathobiology, progressive vascular remodeling, RV consequences, and the distinction between established therapeutic pathways and investigational targets.

## 4. Contemporary Risk-Based Treatment: From Three to Four Therapeutic Pathways

Contemporary pulmonary arterial hypertension (PAH) management is founded on early combination therapy, repeated multiparametric risk assessment, and timely treatment escalation when therapeutic goals are not achieved. The principal objective is to attain and maintain a low-risk profile while preventing clinical deterioration and preserving right ventricular (RV) function [1,2]. The incorporation of bone morphogenetic protein (BMP)/activin-directed treatment has expanded the therapeutic landscape beyond the three traditional vasoactive pathways. However, the availability of an additional mechanism does not eliminate the need for individualized risk assessment or establish that increasing the number of medications necessarily improves outcomes.

### 4.1. Risk Stratification as the Basis for Treatment Decisions

Risk assessment provides the foundation for treatment selection and longitudinal management in PAH. At diagnosis, comprehensive evaluation incorporates clinical characteristics, World Health Organization functional class (WHO-FC), exercise capacity, circulating biomarkers, RV imaging, and invasive hemodynamics [1]. During follow-up, the four-strata risk model classifies patients as low, intermediate-low, intermediate-high, or high risk using WHO-FC, six-minute walk distance (6MWD), and brain natriuretic peptide (BNP) or N-terminal pro-BNP (NT-proBNP) concentrations [1,15].

The four-strata approach was developed to improve prognostic discrimination among patients previously grouped within the broad intermediate-risk category. In the COMPERA registry, analysis of 1655 patients demonstrated improved discrimination of clinical risk and sensitivity to changes in risk status during follow-up [16]. Its prognostic utility was subsequently supported by findings from the French Pulmonary Hypertension Registry [17]. These observations establish intermediate-low and intermediate-high risk as clinically meaningful categories that may inform the urgency and intensity of treatment escalation.

Nevertheless, risk stratification should not be interpreted as a purely numerical exercise. Patients may exhibit discordant findings, such as preserved exercise capacity despite significant RV dysfunction, or elevated natriuretic peptides influenced by comorbid conditions. Consequently, changes in WHO-FC, 6MWD, and biomarkers should be interpreted alongside disease trajectory, RV imaging, hemodynamics, comorbidities, and treatment tolerance [1,2,15]. Invasive reassessment may be particularly valuable when uncertainty regarding disease severity would materially alter treatment decisions.

### 4.2. Initial Combination Therapy

The transition from sequential monotherapy toward early combination treatment was supported by randomized evidence demonstrating the benefits of targeting complementary pathways. In the AMBITION trial, initial ambrisentan plus tadalafil reduced the risk of clinical-failure events by approximately 50% compared with pooled monotherapy among treatment-naïve patients with WHO-FC II or III PAH [18].

For many newly diagnosed patients without high-risk features or substantial cardiopulmonary comorbidities, contemporary treatment algorithms favor initial dual oral therapy combining an endothelin receptor antagonist (ERA) with a phosphodiesterase type 5 inhibitor (PDE5i) [1,2]. Patients presenting with high-risk disease generally require more intensive treatment, including intravenous or subcutaneous prostacyclin therapy in combination with an ERA and PDE5i, when appropriate. Such patients should also undergo early assessment for lung transplantation when clinically indicated [1,2].

Importantly, the benefits of combination treatment depend on the specific intervention rather than the number of agents administered. In TRITON, initial triple oral therapy with macitentan, tadalafil, and selexipag did not significantly improve the primary pulmonary vascular resistance (PVR) endpoint at week 26 compared with macitentan and tadalafil alone, despite substantial PVR reductions in both groups [19]. This finding illustrates why mechanistic complementarity must be supported by demonstrated incremental clinical benefit rather than assumed from pathway coverage alone.

### 4.3. Follow-Up Treatment According to Residual Risk

Following treatment initiation, repeated risk assessment is essential to determine whether the therapeutic response is adequate. Patients who achieve and maintain low-risk status generally continue effective treatment with ongoing surveillance, whereas persistent intermediate or high risk warrants consideration of treatment intensification [1,2,15].

The distinction between intermediate-low and intermediate-high risk is particularly important because these categories represent different prognostic profiles and may justify different approaches to escalation. Patients at intermediate-low risk may be considered for additional evidence-supported therapies, whereas intermediate-high or high-risk disease requires more urgent reassessment of treatment adequacy and consideration of intensive combination treatment, including parenteral prostacyclin therapy [1,2]. Persistent intermediate-high or high-risk status despite optimized therapy should also prompt timely evaluation for lung transplantation in eligible patients.

Treatment decisions should account for the patient’s clinical trajectory rather than risk category alone. Progressive symptoms, declining exercise capacity, worsening RV function, adverse hemodynamic changes, or recurrent PAH-related hospitalization may indicate clinically important deterioration even when a simplified risk score appears relatively stable. Conversely, comorbidities and treatment-related adverse effects may complicate interpretation of individual risk variables.

Thus, risk-guided escalation requires integration of prognostic assessment with clinical judgment, treatment tolerability, and the anticipated benefit of a specific intervention. This approach is particularly relevant as the therapeutic landscape expands to include agents with distinct mechanisms, adverse-effect profiles, and evidence bases.

### 4.4. Emergence of a Fourth Therapeutic Pathway

The treatment framework developed following the 7th World Symposium on Pulmonary Hypertension recognizes four therapeutically actionable pathways: endothelin, nitric oxide, prostacyclin, and BMP/activin signaling [2]. Sotatercept, an activin signaling inhibitor, differs mechanistically from conventional vasoactive therapies by modulating growth-regulatory signaling implicated in pulmonary vascular remodeling.

Randomized clinical trials have demonstrated clinically meaningful benefits of sotatercept when added to background PAH therapy, supporting its incorporation into contemporary management. However, these benefits should be interpreted within the populations, endpoints, and follow-up periods studied. Sotatercept is not a replacement for established vasoactive treatment, and its clinical efficacy does not independently establish histological reversal of pulmonary vascular remodeling or durable disease modification.

The 2026 European Respiratory Society (ERS) guideline update recommends add-on sotatercept for patients receiving PAH therapy who remain at intermediate-low, intermediate-high, or high risk of death during follow-up [20]. No recommendation was made for patients at low risk because of insufficient evidence in this population. Importantly, the absence of a recommendation for low-risk patients should not be interpreted as a recommendation against treatment [20].

The same guideline update addresses follow-up right-heart catheterization in patients with persistent intermediate or high risk when invasive hemodynamic information is expected to influence therapeutic management [20]. The decision to perform invasive reassessment should therefore be individualized according to the clinical question, available noninvasive findings, procedural considerations, and potential consequences for treatment selection.

The introduction of sotatercept also creates additional safety and treatment-sequencing considerations. Hematologic abnormalities, bleeding-related adverse events, vascular adverse effects, and reproductive precautions require appropriate assessment and monitoring. These issues are particularly relevant in patients receiving complex background therapy, including parenteral prostacyclin or antithrombotic treatment. Detailed safety considerations are addressed in Section 9.

### 4.5. Integrating Mechanistically Complementary Treatment into Clinical Practice

The emergence of BMP/activin-directed treatment shifts the therapeutic question from whether additional treatment can be administered to whether a particular intervention offers meaningful incremental benefit for a patient with persistent residual risk.

Four considerations are especially important when evaluating treatment escalation: the patient’s current risk and clinical trajectory; the quality and applicability of evidence supporting the proposed intervention; the anticipated balance between efficacy, adverse effects, and treatment burden; and the extent to which the intervention complements existing therapy.

These considerations are particularly relevant in patients with discordant risk indicators, significant comorbidities, advanced RV dysfunction, or limitations in treatment tolerability. However, individualized judgment must remain anchored to established treatment recommendations. In particular, the availability of sotatercept should not be interpreted as justification for delaying indicated parenteral prostacyclin treatment or lung transplantation evaluation in patients with advanced disease.

Optimal sequencing between established vasoactive therapies and BMP/activin-directed treatment remains incompletely defined. Current evidence supports risk-guided addition of sotatercept in appropriate patients, but it does not establish that a particular sequence of mechanistically complementary agents is universally superior. Similarly, substantial treatment responses do not yet justify routine withdrawal or de-escalation of effective background therapy.

The evolution of PAH treatment from pathway-specific pharmacotherapy toward risk-guided mechanistic diversification is summarized in Table 1.

### 4.6. What This Review Adds Beyond Contemporary Guidelines

The World Symposium treatment framework and the 2026 ERS guideline update provide evidence-based recommendations regarding risk assessment and therapeutic escalation [2,20]. The purpose of the present review is not to replace these recommendations or introduce an independently validated treatment algorithm. Rather, it provides an interpretative framework for evaluating how remodeling-directed therapies may be incorporated into increasingly complex clinical decisions.

The proposed framework distinguishes three levels of therapeutic decision-making: guideline-supported interventions, for which clinical evidence and formal recommendations are available; individualized clinical considerations, in which discordant risk indicators, comorbidities, treatment tolerance, and disease trajectory influence application of established recommendations; and investigational strategies, for which mechanistic rationale or preliminary clinical findings remain insufficient to support routine use.

Within these levels, the review emphasizes evidence maturity, demonstrated incremental benefit, safety, and the distinction between sustained disease control and unproven biological disease modification. This approach is intended to clarify unresolved questions concerning patient selection, therapeutic sequencing, and the interpretation of treatment response rather than establish a new standard of care.

## 5. From Disease Control to Disease Modification

The emergence of therapies targeting biological pathways implicated in pulmonary vascular remodeling has renewed interest in whether pulmonary arterial hypertension (PAH) treatment can progress beyond disease control toward disease modification. However, symptomatic improvement, sustained disease control, and disease modification represent distinct concepts requiring different levels of evidence. Improvements in exercise capacity, hemodynamics, biomarkers, right ventricular (RV) function, or clinical outcomes may demonstrate substantial therapeutic benefit without necessarily establishing reversal of the underlying pulmonary vascular pathology [6,21].

### 5.1. Defining Disease Modification in PAH

Disease modification may be understood as a treatment-induced alteration in the biological processes responsible for disease progression that results in a meaningful and durable change in the expected disease trajectory [6]. In PAH, demonstrating such an effect is challenging because pulmonary vascular lesions cannot generally be assessed serially in living patients, and improvements in clinical status may arise through mechanisms other than structural vascular reversal.

Importantly, a remodeling-directed mechanism of action does not itself establish disease modification. A treatment that influences proliferation, growth-factor signaling, inflammation, or fibrosis may have a compelling biological rationale but must still demonstrate clinically meaningful and durable effects. Conversely, conventional vasoactive therapies may favorably influence long-term outcomes by reducing pulmonary vascular load and preventing RV deterioration, even without direct evidence of histological reverse remodeling.

Accordingly, disease modification should be regarded as an evidence-dependent conclusion rather than a label assigned according to pharmacological mechanism.

### 5.2. Limitations of Conventional Clinical Endpoints

Commonly used PAH endpoints evaluate different aspects of disease activity and therapeutic response. Six-minute walk distance (6MWD) and World Health Organization functional class (WHO-FC) reflect functional status but may be influenced by age, comorbidities, musculoskeletal limitations, and patient effort. Pulmonary vascular resistance (PVR) measures pulmonary vascular load but cannot distinguish reversible vasomotor effects from structural vascular changes. BNP and NT-proBNP provide prognostic information but primarily reflect myocardial stress rather than pulmonary vascular histology [6,14,21].

Cardiac imaging offers complementary information regarding RV size, function, and adaptation to changes in afterload. Clinical-worsening endpoints capture consequential disease progression, although composite outcomes require careful examination of their individual components.

No single measure independently demonstrates biological reversal of pulmonary vascular remodeling. Even concordant improvement across several domains may reflect highly effective disease control rather than disease modification. A stronger assessment therefore requires longitudinal integration of clinical events, hemodynamics, RV imaging, and biological measurements, with recognition of the limitations of each domain.

### 5.3. Durability and Alteration of Disease Trajectory

Durability is central to evaluating potential disease modification. Sustained improvement during continued therapy establishes the persistence of clinical benefit but does not necessarily demonstrate that the underlying disease process has been permanently altered.

Ideally, evidence supporting disease modification would include a durable reduction in clinically important progression, favorable longitudinal changes in pulmonary vascular and RV biology, and reproducible evidence of biological target engagement or structural remodeling. Such findings would need to demonstrate a change in disease trajectory rather than a temporary physiological response [6,21].

Persistence of benefit after treatment withdrawal could provide additional mechanistic evidence, but withdrawal studies may be ethically inappropriate in patients with progressive, potentially life-threatening PAH. Therefore, discontinuation of effective therapy should not be required or recommended solely to investigate disease modification.

Future studies should prioritize longer-term event-based outcomes, serial RV assessment, validated biological or imaging markers, and study designs capable of distinguishing sustained clinical control from alteration of the underlying disease process.

### 5.4. Does Sotatercept Establish Disease Modification?

Sotatercept provides the strongest clinical evidence to date that targeting a pathway associated with pulmonary vascular remodeling can generate meaningful benefit beyond established PAH therapy. Its clinical development program has demonstrated improvements in hemodynamic, functional, biomarker, and clinical-outcome measures across different studied populations [4,5,22,23,24,25].

Nevertheless, the available clinical evidence does not definitively establish disease modification. Histological reversal of human pulmonary vascular lesions has not been demonstrated, validated noninvasive markers of vascular reverse remodeling remain limited, and persistence of benefit after treatment discontinuation is unknown [6,21].

The most defensible interpretation is therefore that sotatercept represents an established, mechanistically distinct treatment with demonstrated incremental clinical efficacy, while its capacity to produce durable biological disease modification remains an important research question.

### 5.5. An Operational Evidence Framework for Evaluating Disease Modification

To improve conceptual clarity, this review proposes a three-level framework for interpreting treatment responses. The framework is intended to organize evidence rather than function as a validated diagnostic classification or clinical treatment algorithm. To improve conceptual clarity, this review proposes a three-level framework for interpreting treatment responses. The framework is intended to organize evidence rather than function as a validated diagnostic classification or clinical treatment algorithm Table 2.

This hierarchy provides an explicit basis for distinguishing clinical efficacy from claims of disease modification. Importantly, the proposed levels should not be interpreted as mandatory sequential stages or prospectively validated thresholds. Rather, they highlight the increasing evidentiary requirements associated with stronger claims regarding alteration of the PAH disease process.

## 6. Activin Signaling Inhibition: The First Clinical Test of a Remodeling-Directed Strategy

Sotatercept represents a major advance in pulmonary arterial hypertension (PAH) treatment because it targets signaling mechanisms implicated in pulmonary vascular remodeling rather than acting primarily through the traditional vasoactive pathways. Its development program has progressed from hemodynamic proof of concept to randomized evidence of functional improvement and reduced clinical worsening across distinct PAH populations.

However, the PULSAR, SPECTRA, STELLAR, ZENITH, and HYPERION studies differ substantially in baseline risk, disease duration, background treatment, primary endpoints, and follow-up. Their findings must therefore be interpreted according to individual trial designs rather than as directly comparable estimates of treatment efficacy.

### 6.1. Therapeutic Rationale

Sotatercept is a recombinant fusion protein comprising the extracellular domain of activin receptor type IIA linked to the Fc domain of human immunoglobulin G1. It acts as a ligand trap for selected transforming growth factor-β (TGF-β) superfamily ligands, including activins and growth differentiation factors, thereby modulating signaling pathways associated with vascular cell growth and remodeling [10,22].

The therapeutic rationale is based on the imbalance between protective bone morphogenetic protein (BMP)-associated signaling and activin/TGF-β-associated signaling observed in PAH. By sequestering selected ligands, sotatercept may attenuate signaling associated with abnormal vascular proliferation. However, it does not directly restore BMPR2 receptor function, and the molecular consequences of treatment are more complex than a simple normalization of SMAD signaling.

Preclinical studies have demonstrated improvements in pulmonary vascular remodeling, hemodynamics, and right ventricular (RV) structure and function following activin-ligand sequestration [26]. Additional experimental investigations have identified effects on inflammatory signaling and pulmonary vascular gene-expression profiles, with some models demonstrating persistent benefits after treatment withdrawal [27].

These findings support a remodeling-directed mechanism but cannot establish equivalent structural or disease-modifying effects in humans. The principal clinical question is whether targeting this pathway produces meaningful incremental benefit beyond established PAH therapy.

### 6.2. PULSAR: Hemodynamic Proof of Concept

PULSAR was a phase II randomized, placebo-controlled trial involving 106 adults with World Health Organization functional class (WHO-FC) II or III PAH receiving stable background therapy [22]. Participants received sotatercept at 0.3 mg/kg or 0.7 mg/kg every three weeks or placebo for 24 weeks.

Both sotatercept doses significantly improved the primary endpoint of pulmonary vascular resistance (PVR). Placebo-corrected changes were −145.8 dyn·s·cm^−5^ with 0.3 mg/kg and −239.5 dyn·s·cm^−5^ with 0.7 mg/kg. Improvements in six-minute walk distance (6MWD) and NT-proBNP provided additional evidence of treatment activity [22].

The subsequent open-label extension demonstrated maintenance of favorable hemodynamic and clinical measures among participants continuing treatment, while patients initially assigned to placebo improved after receiving sotatercept [23].

Nevertheless, PULSAR was relatively small and primarily designed to evaluate short-term hemodynamic efficacy. The uncontrolled extension supports durability during continued treatment but cannot determine whether treatment permanently modifies the underlying disease process.

### 6.3. SPECTRA: Exercise Physiology and Right Ventricular Response

SPECTRA was an exploratory phase IIa study examining physiological responses to sotatercept using invasive cardiopulmonary exercise testing and cardiac magnetic resonance imaging [24].

Treatment was associated with improvements in exercise physiology, including peak oxygen uptake and exercise hemodynamics, alongside favorable changes in measures of RV performance.

These observations provide complementary physiological evidence beyond resting PVR. However, the exploratory design, limited sample size, and absence of a concurrent randomized control group restrict causal interpretation. Improvements in RV performance may also reflect reduced pulmonary vascular afterload rather than a direct myocardial effect.

SPECTRA therefore provides mechanistic and physiological support but should not be interpreted as confirmatory evidence of RV-specific treatment efficacy or structural disease modification.

### 6.4. STELLAR: Phase III Confirmation of Multidomain Clinical Efficacy

STELLAR randomized 323 adults with WHO-FC II or III PAH receiving stable background therapy to sotatercept or placebo [4]. Approximately 61% were receiving triple background therapy, and approximately 40% were receiving prostacyclin infusion treatment.

At week 24, the median change in 6MWD was 34.4 m with sotatercept compared with 1.0 m with placebo, corresponding to a Hodges–Lehmann treatment difference of 40.8 m [4].

Eight of nine hierarchically tested secondary endpoints also showed statistically significant improvements, including measures of hemodynamics, biomarkers, functional status, clinical worsening, and risk-related outcomes.

STELLAR established the efficacy of sotatercept as an add-on treatment in a population receiving substantial background therapy. Its multidomain findings strengthened the evidence that activin signaling inhibition provides benefits beyond those achieved with established treatment alone.

However, the primary assessment occurred at 24 weeks, and the study was not designed to demonstrate histological reverse remodeling, treatment-independent durability, or the optimal sequence of sotatercept relative to other PAH therapies.

### 6.5. ZENITH: Clinical Outcomes in Advanced High-Risk PAH

ZENITH evaluated sotatercept in 172 patients with advanced PAH at high predicted risk of death despite maximally tolerated background therapy [5]. Unlike PULSAR and STELLAR, which emphasized hemodynamic and functional endpoints, ZENITH evaluated a clinically consequential composite endpoint comprising death from any cause, lung transplantation, or hospitalization for worsening PAH.

The trial was stopped early for efficacy following a prespecified interim analysis. A primary endpoint event occurred in 15 of 86 patients (17.4%) receiving sotatercept compared with 47 of 86 (54.7%) receiving placebo, corresponding to a hazard ratio of 0.24 (95% confidence interval [CI], 0.13–0.43) [5].

Importantly, the individual components require separate consideration. PAH-related hospitalization occurred in 8 patients (9.3%) receiving sotatercept and 43 patients (50.0%) receiving placebo. Across the reported component outcomes, all-cause death occurred in 7 (8.1%) versus 13 (15.1%), and lung transplantation in 1 (1.2%) versus 6 (7.0%), respectively. These component totals should not be added together because patients could experience more than one event.

The substantial difference in hospitalization contributed importantly to the composite treatment effect. Although the findings are clinically significant, the composite hazard ratio must not be interpreted as demonstrating an identical reduction in each individual component or definitive independent mortality benefit.

Early termination also limits the precision of long-term safety and individual outcome estimates. Furthermore, ZENITH does not establish that sotatercept can replace parenteral prostacyclin treatment or delay lung transplantation evaluation in advanced disease.

### 6.6. HYPERION: Clinical Benefit Earlier in the Disease Course

HYPERION extended the sotatercept clinical program to adults diagnosed with PAH less than one year earlier who remained at intermediate or high risk despite dual or triple background treatment [25].

The phase III trial randomized 320 patients, with a median follow-up of 13.2 months. The primary endpoint was time to first clinical worsening, comprising death from any cause, hospitalization for worsening PAH, atrial septostomy, lung transplantation, or deterioration in exercise-test performance attributable to PAH.

A primary endpoint event occurred in 17 of 160 patients (10.6%) receiving sotatercept compared with 59 of 160 (36.9%) receiving placebo, corresponding to a hazard ratio of 0.24 (95% CI, 0.14–0.41; *p* < 0.001) [25].

The individual components demonstrate why composite outcomes require careful interpretation. Exercise-test deterioration occurred in 8 patients (5.0%) receiving sotatercept versus 46 (28.8%) receiving placebo, while hospitalization occurred in 3 (1.9%) versus 14 (8.8%). All-cause death occurred in 7 patients (4.4%) receiving sotatercept and 6 (3.8%) receiving placebo. No lung transplantation or atrial septostomy events occurred.

Thus, the overall clinical-worsening benefit was principally reflected in fewer nonfatal worsening events, particularly exercise deterioration, rather than a demonstrated reduction in mortality.

HYPERION was stopped early because positive results from other sotatercept trials led to loss of clinical equipoise. Although the findings support benefit earlier in the disease course, premature termination and differences in endpoint composition limit comparisons with ZENITH and conclusions regarding long-term outcomes.

### 6.7. Critical Cross-Trial Interpretation

The sotatercept program provides consistent evidence of clinical activity across different PAH populations, but the trials should not be considered interchangeable.

PULSAR established hemodynamic efficacy over 24 weeks, while SPECTRA explored exercise physiology and RV responses. STELLAR confirmed functional and multidomain benefits in patients receiving established background therapy. ZENITH evaluated major morbidity and mortality events in advanced high-risk disease, whereas HYPERION investigated clinical worsening earlier after diagnosis.

Differences in disease duration, baseline functional status, background prostacyclin use, primary endpoints, and follow-up duration are important when interpreting the findings. In particular, the composite endpoints in ZENITH and HYPERION differ in their component definitions and observed event distributions.

ZENITH showed a particularly large difference in PAH-related hospitalization, while the HYPERION composite was substantially influenced by exercise-test deterioration. Therefore, the identical reported hazard ratios of 0.24 should not be interpreted as evidence of identical clinical effects or equivalent mortality reductions.

Moreover, both trials ended early, restricting long-term interpretation. Cross-trial comparisons remain descriptive and hypothesis-generating rather than valid estimates of relative efficacy. Moreover, both trials ended early, restricting long-term interpretation. Cross-trial comparisons remain descriptive and hypothesis-generating rather than valid estimates of relative efficacy. A comparative summary of the major sotatercept clinical trials is presented in Table 3.

### 6.8. Safety and Clinical Interpretation

Across the sotatercept development program, important adverse events have included epistaxis, telangiectasia, increased hemoglobin concentrations, thrombocytopenia, and bleeding-related complications [4,5,22,23,25]. These findings underscore the importance of hematologic monitoring, bleeding assessment, and individualized evaluation of treatment tolerability, particularly in patients receiving complex background regimens.

The clinical evidence establishes sotatercept as an effective add-on therapy targeting a pathway associated with pulmonary vascular remodeling. However, it does not establish optimal treatment sequencing, routine withdrawal of existing therapy, or definitive reversal of pulmonary vascular pathology.

Detailed safety monitoring, treatment interruption considerations, reproductive precautions, and interactions with background treatment are discussed in Section 9.

The broader significance of the sotatercept program is that remodeling-associated signaling has become a clinically actionable therapeutic target. Whether other biological approaches can reproduce similarly robust outcomes remains uncertain and must be evaluated according to their individual evidence maturity.

## 7. Beyond Sotatercept: Emerging Strategies Targeting Pulmonary Vascular Remodeling

The clinical success of activin signaling inhibition has strengthened interest in therapies targeting additional biological mechanisms involved in pulmonary vascular remodeling. However, the translation of mechanistic discoveries into effective treatments remains challenging. Several investigational approaches have demonstrated promising preclinical or early clinical findings without subsequently establishing sufficient efficacy or safety for routine clinical use.

A central distinction is therefore required between biological plausibility, evidence of target engagement, preliminary clinical activity, and confirmatory therapeutic efficacy. Unlike sotatercept, which has demonstrated benefits in randomized phase III trials, the strategies discussed below remain investigational. Their potential clinical roles must be evaluated according to evidence maturity, treatment-related toxicity, and demonstrated incremental benefit beyond contemporary background therapy [21].

### 7.1. Growth-Factor Signaling and Tyrosine Kinase Inhibition

Platelet-derived growth factor (PDGF) signaling contributes to pulmonary arterial smooth muscle cell proliferation, migration, and survival, making it an attractive therapeutic target. However, clinical experience with kinase inhibition demonstrates that favorable effects on pulmonary vascular biology do not necessarily translate into an acceptable benefit–risk profile.

In the phase III IMPRES trial, imatinib added to established PAH therapy improved exercise capacity and pulmonary hemodynamics in patients with advanced disease. Nevertheless, treatment was associated with substantial discontinuation and serious adverse events, including subdural hematomas, particularly in the context of anticoagulant exposure [28]. These safety concerns substantially limited its clinical applicability and highlighted the challenges associated with systemic kinase inhibition.

Seralutinib, an inhaled inhibitor of platelet-derived growth factor receptors α and β (PDGFRα/β), colony-stimulating factor 1 receptor (CSF1R), and c-KIT, was developed to target proliferative and inflammatory signaling while potentially reducing systemic exposure. In the randomized phase II TORREY trial, seralutinib significantly improved the primary endpoint of pulmonary vascular resistance (PVR) compared with placebo at 24 weeks in patients receiving background PAH therapy [29]. However, hemodynamic improvement alone was insufficient to establish clinically meaningful long-term efficacy.

The subsequent phase III PROSERA trial enrolled 390 patients with World Health Organization functional class (WHO-FC) II or III PAH receiving background therapy [30]. The primary endpoint was change in six-minute walk distance (6MWD) from baseline to week 24. According to sponsor-reported topline results released in February 2026, seralutinib produced a placebo-adjusted improvement of 13.3 m (*p* = 0.0320), which did not meet the prespecified statistical significance threshold of α = 0.025 [31].

In a prespecified intermediate- and high-risk subgroup, the sponsor reported a placebo-adjusted improvement of 20.0 m (nominal *p* = 0.0207) [31]. Although this finding suggests possible heterogeneity of treatment response, it remains exploratory because the overall primary endpoint did not satisfy the prespecified statistical criterion. Favorable secondary or subgroup findings therefore cannot independently establish confirmatory efficacy following failure of the primary statistical test.

The PROSERA findings illustrate an important distinction between a numerical treatment signal and a statistically confirmed clinical benefit. They also demonstrate why positive phase II hemodynamic findings cannot be assumed to predict phase III success on functional or clinical endpoints. Pending a complete peer-reviewed assessment of efficacy, safety, and subgroup findings, seralutinib should remain classified as investigational rather than an established PAH treatment.

Taken together, the imatinib and seralutinib programs support the biological relevance of growth-factor signaling but demonstrate different translational barriers: systemic toxicity in the former and unconfirmed phase III primary efficacy in the latter.

### 7.2. Restoring Protective BMP/BMPR2 Signaling

Dysfunction of bone morphogenetic protein receptor type 2 (BMPR2) signaling is strongly implicated in PAH pathogenesis, particularly in heritable disease. Strategies designed to restore protective bone morphogenetic protein (BMP) signaling therefore represent a mechanistically distinct approach from ligand sequestration with sotatercept.

Potential interventions include pharmacological enhancement of BMPR2 signaling, restoration of downstream signaling activity, and gene-based delivery of functional BMPR2 [3,9,10,32,33].

Preclinical investigations demonstrated that low-dose tacrolimus could activate BMPR2-associated signaling and improve experimental pulmonary hypertension [32]. This approach subsequently progressed to a randomized, double-blind, placebo-controlled phase IIa study involving 23 patients with stable WHO-FC II or III PAH. Low-level tacrolimus exposure was generally tolerated during the 16-week treatment period, but the study was primarily designed to assess safety and feasibility rather than establish clinical efficacy [34].

Consequently, these findings should not be interpreted as evidence supporting routine tacrolimus use in PAH.

Gene-based BMPR2 restoration has also demonstrated encouraging results in experimental models, including attenuation of pulmonary vascular remodeling and hemodynamic abnormalities [33]. However, translation into clinical treatment faces important challenges, including efficient and selective pulmonary vascular delivery, sustained transgene expression, immunogenicity, genetic heterogeneity, and long-term safety.

Importantly, the success of sotatercept does not establish equivalent efficacy for direct BMPR2 activation or gene replacement. Although these strategies involve related biological pathways, they differ substantially in mechanism, delivery requirements, clinical maturity, and potential adverse effects.

Direct restoration of protective BMP/BMPR2 signaling therefore remains a promising but predominantly preclinical or early clinical therapeutic direction.

### 7.3. Immune and Metabolic Modulation

Inflammatory, immune, and metabolic abnormalities are increasingly recognized as contributors to PAH pathobiology. However, their heterogeneity across disease etiologies and individual patients complicates therapeutic translation.

Immune-directed interventions may be particularly relevant in selected patients with autoimmune-associated PAH. In a randomized, double-blind, placebo-controlled proof-of-concept trial involving 57 patients with systemic sclerosis-associated PAH receiving stable background treatment, rituximab produced a numerical improvement in 6MWD at 24 weeks, but the primary analysis did not reach statistical significance (*p* = 0.12) [35].

An analysis incorporating observations through 48 weeks suggested a possible treatment effect, while exploratory biomarker analyses identified lower baseline rheumatoid factor, interleukin-12, and interleukin-17 levels as potential predictors of response [35]. However, these findings require prospective confirmation and should not be interpreted as demonstrating established PAH efficacy.

Metabolic modulation represents another investigational strategy. In a single-center, open-label phase II study involving 20 patients with idiopathic or heritable PAH, metformin was generally well tolerated but did not produce a significant improvement in 6MWD [36].

Exploratory analyses identified favorable changes in right ventricular (RV) fractional area change and myocardial triglyceride content in a limited subset of participants. However, the small sample size, uncontrolled design, and exploratory nature of these findings preclude conclusions regarding clinical efficacy or direct myocardial benefit.

These studies illustrate the importance of distinguishing biological activity from patient-important outcomes. The presence of immune or metabolic abnormalities does not necessarily indicate that they represent the dominant therapeutic mechanism in every patient.

Future development may therefore benefit from biomarker-guided enrichment strategies capable of identifying biologically responsive subgroups. However, such approaches require prospective validation before they can guide routine treatment selection.

### 7.4. Extracellular Matrix, Mechanotransduction, Epigenetic, and Other Remodeling Targets

Extracellular matrix remodeling, vascular stiffness, mechanotransduction, and epigenetic dysregulation represent additional potential therapeutic domains in PAH. Experimental investigations have identified interactions among matrix-dependent signaling, cellular metabolism, and vascular proliferation, while broader discussions of emerging therapeutic targets have highlighted their potential translational relevance [3,11,12,37,38].

Altered collagen deposition, matrix organization, and vascular stiffness can influence pulmonary vascular cell proliferation, migration, metabolism, and survival. These changes may contribute to reciprocal interactions in which structural remodeling modifies cellular signaling, while activated vascular cells promote further matrix production.

Experimental studies have implicated yes-associated protein/transcriptional coactivator with PDZ-binding motif (YAP/TAZ)-dependent signaling in this process. Increased matrix stiffness can promote metabolic reprogramming, including glutaminolysis, supporting the biosynthetic requirements of proliferating pulmonary vascular cells [11,38].

Additional experimental evidence has linked glutamine and serine metabolism in pulmonary arterial adventitial fibroblasts to collagen biosynthesis and vascular stiffening [12]. Pharmacological or dietary interventions targeting these processes have demonstrated favorable effects in experimental systems, providing proof of biological relevance.

However, these findings remain predominantly preclinical. The extent to which targeting matrix stiffness, mechanotransduction, or associated metabolic pathways will produce clinically meaningful benefits in human PAH remains uncertain.

Epigenetic and transcriptional abnormalities may also contribute to the persistence of proliferative, apoptosis-resistant, inflammatory, and fibrotic vascular phenotypes. These mechanisms have been discussed as potential therapeutic targets in the emerging PAH treatment landscape [37]. However, the cited overview is an editorial rather than primary clinical efficacy evidence, and the clinical development of these approaches remains insufficient to establish their therapeutic benefit, long-term safety, or optimal patient selection.

These pathways should therefore be considered mechanistic research priorities rather than clinically established therapeutic options.

### 7.5. Evidence Maturity and the Prospects for Precision Remodeling Therapy

The emerging therapeutic landscape encompasses interventions at substantially different stages of development. Their clinical implications cannot be assessed solely according to the biological pathways they target.

Systemic tyrosine kinase inhibition has demonstrated randomized clinical efficacy signals but encountered important toxicity limitations. Inhaled seralutinib has progressed to phase III evaluation without meeting its prespecified primary statistical criterion. Pharmacological BMPR2 activation and immune or metabolic modulation have undergone limited early clinical testing, while gene-based restoration, extracellular matrix targeting, and mechanotransduction-directed interventions remain largely preclinical.

These differences support the need for an explicit evidence-maturity framework, summarized in Table 4.

The substantial variation in evidence maturity has implications for future therapeutic development. Biomarkers, genetic profiling, molecular phenotyping, imaging, and functional measurements may eventually help identify patients most likely to respond to particular remodeling-directed interventions [21].

However, most proposed biological markers have not been prospectively validated for selecting remodeling-directed treatment. A biomarker associated with disease severity or pathway activity does not necessarily predict treatment response.

Future trials should therefore evaluate whether biomarker-defined enrichment strategies improve treatment effects, reduce unnecessary exposure, or identify clinically meaningful response heterogeneity. Such investigations should incorporate clinically relevant endpoints, safety assessments, and comparison with contemporary risk-guided management.

Precision remodeling therapy remains a research objective rather than an established treatment paradigm. Its successful development will require demonstration that biologically informed treatment selection improves patient outcomes beyond those achieved through current risk-based strategies.

## 8. Integrating Remodeling-Directed Therapy into Risk-Guided PAH Management

The incorporation of activin signaling inhibition into pulmonary arterial hypertension (PAH) management raises an important clinical question: how should mechanistically distinct treatment be integrated into existing risk-guided strategies without compromising established standards of care? The 2026 European Respiratory Society (ERS) guideline update recommends add-on sotatercept for patients receiving PAH therapy who remain at intermediate-low, intermediate-high, or high risk during follow-up [20]. Its introduction therefore represents an expansion of evidence-based therapeutic escalation rather than a replacement for conventional combination therapy, parenteral prostacyclin treatment, or lung transplantation assessment [1,2,20].

The framework presented here distinguishes guideline-supported recommendations from individualized clinical considerations and investigational strategies. It is intended to facilitate interpretation of contemporary evidence in complex clinical situations, not to introduce a prospectively validated treatment algorithm.

### 8.1. Initial Therapy and Early Reassessment

For most newly diagnosed patients with idiopathic, heritable, drug-associated, or connective tissue disease-associated PAH who are not at high risk and do not have substantial cardiopulmonary comorbidities, initial combination treatment with an endothelin receptor antagonist (ERA) and phosphodiesterase type 5 inhibitor (PDE5i) remains a principal therapeutic approach [1,2].

Patients presenting with high-risk disease generally require more intensive treatment, including intravenous or subcutaneous prostacyclin therapy as part of combination treatment. Early referral to an expert pulmonary hypertension center and consideration of lung transplantation are important in patients with advanced disease [1,2].

Treatment response should be assessed early and repeatedly using clinical status, exercise capacity, natriuretic peptides, and complementary measures of right ventricular (RV) function and hemodynamics when appropriate.

HYPERION demonstrated that sotatercept can reduce clinical worsening among patients diagnosed within the preceding year who remain at intermediate or high risk despite background treatment [25]. These findings extend the evidence supporting activin signaling inhibition toward earlier disease but do not establish sotatercept as universal treatment-naïve first-line therapy.

The relevant distinction is therefore between earlier evidence-supported escalation in patients with residual risk and indiscriminate initiation of additional treatment irrespective of risk or response.

### 8.2. Treatment According to Follow-Up Risk

Risk assessment provides the principal basis for treatment escalation, but its application must account for clinical trajectory, treatment response, comorbidities, and tolerability.

Low-risk patients. Patients who achieve and maintain low-risk status generally continue effective treatment with longitudinal surveillance [1,2]. The 2026 ERS update did not make a recommendation regarding sotatercept for patients at low risk because evidence was insufficient [20]. This should not be interpreted as a recommendation against sotatercept, nor as support for routine addition in all low-risk patients.

Intermediate-low-risk patients. Persistent intermediate-low risk represents an important opportunity for treatment intensification. Add-on sotatercept is recommended by the 2026 ERS update for patients already receiving PAH therapy who remain within this risk category [20]. However, the selection and implementation of escalation should account for existing treatment, RV function, hemodynamics, comorbidities, bleeding risk, treatment burden, and patient preferences.

Intermediate-high-risk patients. Persistent intermediate-high risk warrants prompt specialist reassessment and consideration of more intensive treatment. Add-on sotatercept is guideline-supported in this population [20], but its incorporation should not displace other indicated escalation strategies. In patients with significant RV dysfunction, clinical deterioration, or concerning hemodynamic findings, parenteral prostacyclin therapy and transplantation assessment remain important considerations [1,2].

High-risk patients. High-risk disease requires urgent expert-center management. Intravenous or subcutaneous prostacyclin-based combination therapy remains central to treatment, and lung transplantation evaluation should be considered without unnecessary delay [1,2]. ZENITH demonstrated additional clinical benefit from sotatercept in advanced high-risk PAH despite substantial background treatment [5]. Nevertheless, this finding supports complementary treatment rather than replacement of established high-risk interventions.

These risk categories should guide treatment decisions without being applied mechanically. Rapid clinical deterioration or major RV dysfunction may require urgent intervention even when simplified risk variables do not fully reflect disease severity.

### 8.3. Reconciling Discordant Risk Indicators

Although WHO functional class, six-minute walk distance (6MWD), and BNP or NT-proBNP are central to follow-up risk assessment, these measures may not always provide a concordant picture of disease severity.

For example, preserved functional capacity may coexist with markedly elevated pulmonary vascular resistance (PVR) or impaired RV function. Conversely, reduced exercise capacity or elevated biomarkers may be influenced by comorbidities unrelated to pulmonary vascular disease.

When findings are discordant, management should begin with reassessment of measurement quality, treatment adherence, comorbidities, volume status, and disease trajectory. Echocardiography, cardiac magnetic resonance imaging, and invasive hemodynamics may provide complementary information when clinically indicated.

The 2026 ERS update suggests follow-up right-heart catheterization in patients at intermediate-low, intermediate-high, or high risk when the findings are expected to influence treatment decisions [20]. Thus, invasive reassessment should address a specific clinical uncertainty rather than be performed solely to satisfy a numerical treatment target. This approach represents individualized clinical interpretation of guideline principles, not a separately validated risk algorithm.

### 8.4. Balancing Efficacy, Safety, and Treatment Burden

The availability of a fourth therapeutic pathway increases the importance of evaluating treatment burden and safety alongside anticipated efficacy. Sotatercept requires repeated subcutaneous administration, hematologic monitoring, and surveillance for adverse effects, including increased hemoglobin concentration, thrombocytopenia, epistaxis, telangiectasia, and bleeding-related complications [4,5,22,23,25].

Patients receiving infused prostacyclin therapy or antithrombotic treatment may require particular attention to bleeding risk. Baseline hematologic abnormalities, prior serious bleeding, reproductive considerations, and the feasibility of laboratory surveillance may also influence treatment planning.

These factors do not necessarily preclude sotatercept treatment but may affect the timing, monitoring intensity, and practical implementation of guideline-supported escalation. They should be considered alongside clinical severity, previous treatment response, and patient preferences.

Importantly, adverse-effect concerns should not lead to unsupported substitution or withdrawal of therapies with established benefit. Decisions regarding treatment modification should be individualized in expert PAH centers. Detailed monitoring and dose-interruption considerations are presented in Section 9.

### 8.5. Mechanistically Complementary Treatment Without Indiscriminate Escalation

The contemporary four-pathway framework permits treatment combinations involving endothelin, nitric oxide–cyclic guanosine monophosphate, prostacyclin, and BMP/activin signaling [2]. However, comprehensive pathway coverage should not be regarded as an independent therapeutic goal.

The TRITON trial demonstrated that increasing the number of pharmacological agents does not necessarily produce additional benefit on a prespecified primary endpoint [19]. Accordingly, treatment escalation should depend on evidence supporting the specific intervention in the relevant patient population.

The principal consideration is whether a proposed treatment provides meaningful incremental benefit in the context of persistent residual risk, while maintaining an acceptable safety and treatment-burden profile.

For sotatercept, randomized evidence and contemporary recommendations support its addition in specified follow-up risk categories. For other remodeling-directed strategies, including kinase inhibition, BMPR2 restoration, immune modulation, and metabolic or extracellular matrix interventions, evidence remains insufficient for routine incorporation into clinical treatment pathways.

Thus, mechanistic complementarity should inform therapeutic development and clinical interpretation but must not substitute for demonstrated efficacy.

### 8.6. Lung Transplantation in the Four-Pathway Era

Advances in pharmacological therapy do not eliminate the role of lung transplantation in advanced PAH. Patients with persistent intermediate-high or high-risk disease despite optimized treatment should undergo timely transplantation evaluation when appropriate [1,2]. In patients with severe disease, pharmacological intensification and transplantation assessment may proceed concurrently rather than sequentially.

This principle is particularly important as additional therapeutic options become available. Repeated attempts to introduce new agents should not create unnecessary delays in referral for patients whose prognosis remains poor despite treatment. Sotatercept may provide additional clinical benefit in advanced disease, but its availability should not be interpreted as justification for postponing indicated parenteral prostacyclin therapy or transplantation evaluation.

### 8.7. Illustrative Clinical Decision Scenarios

The following scenarios demonstrate how guideline recommendations may be interpreted alongside individual clinical considerations. They are hypothetical examples intended to clarify clinical reasoning and do not constitute a validated treatment algorithm.

Scenario 1: Persistent intermediate-low risk despite dual oral therapy

A patient receiving an ERA and PDE5i remains at intermediate-low risk during follow-up, with persistent functional limitation and elevated NT-proBNP but stable RV function and no major bleeding concerns.

Add-on sotatercept is guideline-supported in this setting [20]. The treatment discussion should also consider prior treatment response, existing medication burden, hematologic parameters, monitoring feasibility, and patient preferences.

The guideline-supported element is the recommendation for add-on sotatercept in persistent intermediate-low risk. The individualized element is the assessment of how best to implement escalation in the context of the patient’s existing treatment and safety profile.

Scenario 2: High-risk disease with progressive RV dysfunction

A patient receiving combination PAH therapy develops worsening RV function, recurrent syncope, and persistent high-risk status.

This presentation requires urgent expert-center assessment and intensive treatment, including parenteral prostacyclin-based escalation when indicated, together with timely lung transplantation evaluation [1,2].

Sotatercept may provide complementary benefit in an appropriate patient, but its addition must not delay established interventions for advanced disease [5,20].

Scenario 3: Discordant functional and hemodynamic findings

A patient demonstrates improved 6MWD and WHO functional class after treatment, but follow-up imaging reveals persistent RV enlargement and worsening RV function. Previous invasive assessment also demonstrated substantially elevated PVR.

A favorable simplified risk score should not automatically override concerning objective findings. The appropriate response is to reassess clinical trajectory, comorbidities, treatment adherence, and the reliability of individual measurements.

Repeat right-heart catheterization may be considered when the findings are likely to alter therapeutic management [20]. Any subsequent escalation should be based on integrated clinical assessment rather than an isolated threshold.

Scenario 4: Residual risk with substantial bleeding or hematologic concerns

A patient remains at intermediate-low risk despite background treatment but has thrombocytopenia, recurrent bleeding episodes, or concurrent antithrombotic exposure.

Although sotatercept is guideline-supported for persistent intermediate-low risk, treatment initiation requires careful evaluation of hematologic parameters, bleeding history, background medications, and product-label precautions.

The decision should distinguish the evidence supporting efficacy from patient-specific safety constraints. Where treatment cannot be initiated safely, alternative evidence-based management options and close reassessment should be considered.

These examples illustrate why risk-guided management requires both adherence to evidence-based recommendations and individualized assessment of factors that may influence treatment feasibility.

### 8.8. Proposed Integrated Clinical Interpretation Framework

The incorporation of remodeling-directed therapy can be organized into an iterative process involving initial treatment, reassessment, characterization of residual risk, evidence-supported escalation, and subsequent evaluation of response.

Within this process, three categories of therapeutic decision-making should remain distinct:

1. Guideline-supported management: Initial combination therapy, risk-based escalation, add-on sotatercept in specified follow-up risk groups, indicated parenteral prostacyclin treatment, and timely transplantation assessment.
2. Individualized clinical considerations: Interpretation of discordant risk variables, selection among appropriate escalation options, management of comorbidities, assessment of bleeding risk and treatment burden, and decisions regarding additional hemodynamic evaluation.
3. Investigational approaches: Therapies targeting growth-factor signaling, direct BMPR2 restoration, immune dysregulation, metabolism, extracellular matrix remodeling, or other emerging pathways without sufficient evidence for routine clinical use.

These categories are intended to clarify the evidentiary basis of clinical decisions rather than establish a new treatment hierarchy.

Figure 2 illustrates the integration of established treatment, residual-risk assessment, sotatercept, intensive high-risk management, and investigational therapeutic strategies. Guideline-supported interventions should be visually distinguished from individualized considerations and research-only approaches to prevent the conceptual framework from being mistaken for a validated clinical algorithm.

The central principle is that remodeling-directed therapy should be incorporated according to demonstrated clinical benefit and individual patient risk, rather than added indiscriminately on the basis of mechanistic availability alone.

## 9. Safety, Monitoring, and Practical Considerations

The expansion of pulmonary arterial hypertension (PAH) treatment to include activin signaling inhibition introduces additional safety and monitoring considerations alongside those associated with established vasoactive therapies. As treatment regimens become increasingly complex, therapeutic decisions must account for adverse effects, drug interactions, route of administration, monitoring requirements, reproductive considerations, and the cumulative burden of long-term treatment. Safety assessment should be integrated with evaluation of clinical benefit and residual risk rather than treated as a separate component of care.

### 9.1. Safety Considerations with Established PAH Therapies

Established PAH therapies have distinct adverse-effect profiles that influence treatment selection, escalation, and long-term management.

Endothelin receptor antagonists (ERAs) may cause fluid retention, anemia, hepatic abnormalities, and embryo-fetal toxicity. Monitoring requirements vary among individual agents and should follow the applicable prescribing information. Phosphodiesterase type 5 inhibitors (PDE5is) and soluble guanylate cyclase stimulators may cause headache, systemic hypotension, and other vasodilator-related adverse effects. Concomitant administration of PDE5is and riociguat is contraindicated because of the risk of excessive hypotension [1,2].

Prostacyclin pathway therapies commonly cause headache, flushing, jaw pain, gastrointestinal symptoms, and extremity pain. Continuous intravenous or subcutaneous prostacyclin administration introduces additional treatment-related burdens, including infusion-site reactions, device complications, and, with intravenous treatment, catheter-associated infections. Abrupt interruption of parenteral prostacyclin therapy can precipitate rapid clinical deterioration and must be avoided [2].

The cumulative burden of multidrug treatment is particularly relevant in patients receiving continuous infusion therapy or requiring frequent clinical assessments. Treatment complexity may affect adherence, daily functioning, quality of life, and the feasibility of sustaining therapy over prolonged periods.

Nevertheless, treatment burden should not be considered independently of clinical severity. In patients with advanced PAH, therapies associated with substantial practical demands may remain essential because of their established clinical benefits.

### 9.2. Sotatercept-Specific Safety and Hematologic Monitoring

Sotatercept has a safety profile that differs from conventional vasoactive therapies. Across randomized trials and extension studies, important adverse events have included increased hemoglobin concentration, thrombocytopenia, epistaxis, telangiectasia, and other bleeding-related events [4,5,22,23,25]. Serious hypersensitivity reactions, including anaphylaxis and angioedema, have also been reported during postmarketing use [39].

These findings require particular attention because sotatercept is generally administered in addition to existing PAH treatment, including regimens that may already involve parenteral prostacyclin therapy or antithrombotic medications.

Current U.S. prescribing information requires assessment of hemoglobin and platelet counts before treatment initiation and before each of the first five doses, with continued assessment before subsequent doses if values remain unstable and periodic monitoring thereafter [39]. Monitoring intervals and dose-modification requirements should follow the locally applicable prescribing information.

Sotatercept-associated erythrocytosis may increase the risk of thromboembolic events or hyperviscosity syndrome. Under U.S. labeling, treatment should be delayed for at least three weeks if hemoglobin increases by more than 2 g/dL from the previous dose and exceeds the upper limit of normal, increases by more than 4 g/dL from baseline, or exceeds the upper limit of normal by more than 2 g/dL [39].

Thrombocytopenia may increase bleeding susceptibility, particularly in patients receiving concomitant prostacyclin infusion. Sotatercept should not be initiated in patients with platelet counts below 50,000/mm^3^. A decline below this threshold during treatment also requires a treatment delay of at least three weeks under U.S. labeling [39].

Hemoglobin and platelet counts should be reassessed before treatment is resumed. Following a treatment interruption exceeding nine weeks, U.S. prescribing information recommends restarting at 0.3 mg/kg and subsequently escalating to 0.7 mg/kg after confirming acceptable hematologic values [39]. These recommendations should not be extrapolated to jurisdictions with different approved product information.

Bleeding surveillance should include assessment of epistaxis, gastrointestinal bleeding, bruising, and other clinically significant manifestations. Sotatercept should not be administered during serious bleeding, and the underlying cause should be evaluated and treated [39]. Postmarketing cases of gastrointestinal bleeding associated with angiodysplasias have also been reported, and endoscopic evaluation should be considered in patients with recurrent or unexplained gastrointestinal bleeding [39].

Medication review remains important, particularly in patients receiving antithrombotic therapy or infused prostacyclin. Clinical assessment should consider both laboratory abnormalities and bleeding manifestations, because hematologic monitoring alone does not fully characterize individual bleeding risk.

Serious hypersensitivity reactions require a different management approach from temporary hematologic treatment delays. If a serious hypersensitivity reaction, including anaphylaxis or angioedema, occurs, sotatercept should be discontinued and appropriate emergency treatment initiated [39]. A history of serious hypersensitivity to sotatercept or its excipients is a contraindication under the current U.S. prescribing information [39].

The timing of adverse effects also warrants consideration. In the PULSAR open-label extension, telangiectasia became more apparent with longer treatment exposure, suggesting that short randomized treatment periods may not fully characterize delayed or cumulative adverse events [23].

Longer-term observations from SOTERIA have expanded the available safety experience beyond the pivotal randomized trials [40]. Nevertheless, extension studies and clinical trial populations may not fully reflect patients encountered in routine practice, particularly those with substantial comorbidities, complex medication regimens, or pre-existing hematologic abnormalities.

Continued pharmacovigilance remains necessary to characterize uncommon, cumulative, or delayed adverse events and to determine whether monitoring recommendations require modification with prolonged treatment exposure.

### 9.3. Reproductive Safety and Patient Counseling

Reproductive counseling is an essential component of sotatercept treatment planning.

Based on animal reproductive toxicity findings, sotatercept may cause embryo-fetal harm. Pregnancy testing is recommended before treatment initiation in patients who can become pregnant, and effective contraception should be used during treatment and for at least four months after the final dose, according to U.S. prescribing information [39].

Patients should also be counseled regarding the potential for impaired fertility in both females and males, including uncertainty about the reversibility of reproductive effects. Breastfeeding is not recommended during treatment and for four months after the final dose under U.S. labeling [39].

These precautions should be discussed before treatment initiation and revisited when clinically appropriate. Requirements may differ across jurisdictions, and clinicians should consult the locally approved prescribing information.

Reproductive considerations should be incorporated into shared decision-making alongside the anticipated benefits of treatment, alternative management options, and the risks associated with uncontrolled PAH.

### 9.4. Practical Monitoring and Treatment-Interruption Considerations

The principal safety considerations associated with sotatercept and their practical implications are summarized in Table 5.

### 9.5. Integrating Safety with Treatment Response and Long-Term Management

Safety monitoring should be integrated with repeated assessment of clinical efficacy and residual risk. WHO functional class, exercise capacity, BNP or NT-proBNP, RV function, and other clinically relevant variables should be evaluated alongside adverse effects and treatment tolerability [1,20].

This integrated approach becomes particularly important in patients who experience substantial improvement after treatment escalation. A favorable response may alter the perceived balance between therapeutic benefit and treatment burden, but it does not establish that previously effective background therapy has become unnecessary.

Conversely, clinically significant toxicity, recurrent bleeding, treatment intolerance, or substantial practical burden may require reconsideration of individual treatment components. Such decisions should account for current risk status, disease trajectory, the likely contribution of each treatment to disease control, and the consequences of treatment interruption.

There is currently insufficient prospective evidence to support routine de-escalation of established PAH treatment following improvement with sotatercept or other combination therapies [2,20]. In particular, withdrawal of parenteral prostacyclin treatment may carry substantial risk and should not be inferred to be safe solely because a patient achieves favorable functional, hemodynamic, or biomarker responses.

The possibility of safely reducing treatment burden in carefully selected patients remains an important research question. Future studies should evaluate whether specific clinical, hemodynamic, imaging, or biological characteristics can identify patients in whom treatment simplification may be considered without compromising long-term outcomes.

Until such evidence becomes available, sustained clinical improvement should generally support continued individualized monitoring and evidence-based management rather than automatic treatment withdrawal.

## 10. Clinical Implications and Future Research Priorities

The principal clinical implication of the expanding PAH therapeutic landscape is that treatment escalation can increasingly incorporate mechanisms beyond conventional vasodilation. However, the objective is not universal exposure to every available pathway. Rather, treatment selection should be guided by residual clinical risk, demonstrated incremental efficacy, safety, and the suitability of a particular intervention for the individual patient.

The introduction of sotatercept illustrates this transition. Its randomized clinical evidence supports the therapeutic value of targeting activin signaling in addition to established PAH treatment, while contemporary guidelines provide a framework for its incorporation into risk-based management [2,20]. Nevertheless, optimal treatment sequencing, long-term durability, management of patients with discordant risk indicators, and the implications of profound multidomain responses remain incompletely defined.

The broader challenge is to determine whether treatment selection can eventually progress from risk-guided escalation toward biologically informed precision therapy. This would require identifying not only which patients remain at risk but also which molecular or physiological mechanisms contribute most substantially to their residual disease.

### 10.1. Priority Research Questions

Several unresolved questions should guide future clinical and translational research.

First, biomarker-guided treatment selection requires prospective validation. Although molecular, genetic, inflammatory, metabolic, and imaging biomarkers may help characterize biological heterogeneity, their ability to predict differential responses to remodeling-directed treatment remains uncertain. Prospective studies should determine whether biomarker-informed selection improves outcomes compared with contemporary risk-guided management alone.

Second, optimal treatment sequencing remains undefined. Randomized comparative studies are needed to evaluate the timing and sequence of sotatercept and other established escalation options on standardized background regimens. Such investigations should assess clinical efficacy, adverse effects, treatment burden, and the consequences of delaying therapies with established benefit in advanced disease.

Third, long-term clinical outcomes require continued evaluation. Future studies should prioritize durable event-based endpoints, including clinical worsening, hospitalization, transplantation, and mortality. Composite endpoints should be accompanied by transparent reporting of individual components, absolute event rates, and follow-up duration to support clinically meaningful interpretation.

Fourth, validated measures of pulmonary vascular reverse remodeling are needed. Conventional hemodynamic, functional, biomarker, and RV imaging endpoints cannot independently distinguish reversible physiological improvement from structural alteration of pulmonary vascular disease. Development and validation of biological or imaging measures capable of supporting stronger inferences about remodeling would substantially advance the evaluation of potential disease modification.

Fifth, treatment de-escalation requires rigorous investigation. As deeper treatment responses become achievable, carefully designed studies should determine whether treatment burden can be reduced safely in selected patients. Such research must prioritize patient safety, particularly when evaluating changes to parenteral prostacyclin therapy, and should not assume that sustained improvement permits withdrawal of effective treatment.

### 10.2. Toward Evidence-Calibrated Precision Management

Future PAH management may increasingly integrate conventional clinical risk assessment with molecular phenotyping, genetic information, RV characterization, and biomarkers of treatment response. However, this transition must be supported by prospective evidence demonstrating that biologically informed decisions improve clinically meaningful outcomes.

Until such evidence is available, the distinction between established therapy and investigational mechanisms remains essential. Sotatercept has demonstrated clinical efficacy and has been incorporated into contemporary treatment recommendations for specified risk groups, whereas other remodeling-directed approaches remain at substantially earlier stages of development.

The emerging paradigm is therefore best characterized as risk-guided, evidence-calibrated therapeutic diversification, with precision remodeling therapy representing a future objective rather than an established clinical standard.

This approach preserves the foundations of contemporary PAH management while providing a structured basis for evaluating new therapeutic mechanisms, unresolved clinical decisions, and future opportunities to achieve more durable disease control.

## 11. Future Directions

The next phase of pulmonary arterial hypertension (PAH) research must determine whether the clinical benefits of remodeling-directed treatment can be translated into durable changes in disease trajectory and more precise therapeutic selection. Although activin signaling inhibition has established the clinical relevance of targeting remodeling-associated biology, important uncertainties remain regarding long-term effects, optimal treatment sequencing, biological response heterogeneity, and the interpretation of clinical endpoints [6,21].

Addressing these uncertainties will require research strategies that integrate mechanistic investigations with prospective clinical validation. In particular, future studies should move beyond demonstrating pathway activity toward determining whether biological characteristics can predict differential treatment benefit and improve outcomes beyond established risk-guided management.

### 11.1. Establishing Evidence for Disease Modification

A major research priority is the development of an evidence framework capable of distinguishing sustained clinical disease control from modification of the underlying disease process.

Current PAH trials primarily assess exercise capacity, pulmonary vascular resistance (PVR), biomarkers, right ventricular (RV) function, and clinical outcomes. Although these measures provide important evidence of treatment efficacy, none independently demonstrates reversal of pulmonary vascular pathology [6,21].

Future studies should therefore investigate whether longitudinal changes in pulmonary vascular structure, molecular signaling, and RV adaptation can be reliably measured and associated with meaningful clinical outcomes. Advanced imaging, circulating biomarkers, and molecular measures of target engagement may provide complementary information, but their validity as indicators of structural reverse remodeling must first be established.

Longer-term event-based follow-up will also be necessary to determine whether treatment changes the expected trajectory of clinical deterioration. Importantly, sustained benefit during continued treatment should not automatically be interpreted as evidence of treatment-independent disease modification.

Research should prioritize converging biological and clinical evidence rather than relying on a single surrogate endpoint. Such an approach may strengthen the interpretation of remodeling-directed treatment effects without requiring ethically problematic withdrawal of effective therapy.

### 11.2. Precision Phenotyping and Predictive Biomarkers

PAH is biologically heterogeneous, even among patients with similar clinical or hemodynamic characteristics. Genetic susceptibility, inflammatory signaling, metabolic abnormalities, growth-factor activity, extracellular matrix remodeling, and RV adaptation may vary substantially between individuals [3,9,10,11,12,13,14].

This heterogeneity raises the possibility that different patients may respond preferentially to different remodeling-directed interventions.

Integration of genomic, transcriptomic, proteomic, metabolomic, imaging, and circulating biomarker data may improve characterization of these biological differences. For example, genetic and molecular markers associated with BMPR2 dysfunction may inform future investigations of BMP-directed therapies, while inflammatory or metabolic signatures may help identify candidate populations for immune- or metabolism-directed interventions [9,10,11,12,13,38].

However, identifying a biological abnormality is not equivalent to demonstrating that it predicts treatment response. Biomarkers intended for therapeutic selection must be evaluated for their ability to distinguish patients who derive greater benefit from a specific intervention.

Future studies should therefore incorporate prospective biomarker-treatment interaction analyses, prespecified enrichment strategies, and independent validation cohorts. Where feasible, biomarker-guided approaches should be compared directly with conventional risk-guided treatment selection.

The ultimate objective is to establish whether biological phenotyping provides clinically actionable information beyond existing risk assessment rather than merely improving disease classification.

### 11.3. Optimizing Treatment Timing and Sequencing

The expanding therapeutic landscape introduces uncertainty regarding the optimal timing and sequence of treatment escalation.

HYPERION demonstrated clinical benefit from sotatercept in patients diagnosed with PAH within the preceding year who remained at intermediate or high risk despite background therapy [25]. However, these findings do not establish universal treatment-naïve initiation or identify the optimal sequence of sotatercept relative to other established therapies.

Future randomized studies should evaluate treatment sequencing within clearly defined risk populations and on comparable background regimens. Relevant questions include whether earlier addition of remodeling-directed therapy provides incremental benefit, whether particular clinical phenotypes favor one escalation strategy over another, and how treatment timing influences long-term clinical outcomes.

In intermediate-high- and high-risk disease, such investigations must preserve established standards of care, including appropriate parenteral prostacyclin treatment and timely transplantation assessment [1,2,20]. Trials should not delay effective interventions merely to test alternative treatment sequences.

The opposite question—whether substantial improvement permits safe treatment reduction—also requires investigation. Prospective studies should determine whether any clinical, hemodynamic, imaging, or biological characteristics can identify patients suitable for carefully supervised de-escalation.

Until such evidence is available, treatment simplification, particularly withdrawal of parenteral prostacyclin therapy, should not be assumed to be safe on the basis of favorable clinical responses alone.

### 11.4. Advancing Trial Design and Clinical Endpoints

As PAH treatment becomes more effective, future therapies must demonstrate incremental benefit against increasingly intensive background regimens. This creates important methodological challenges for trial design.

Short-term changes in 6-min walk distance or PVR may remain useful for detecting physiological treatment effects but may be insufficient to establish the long-term clinical value of novel remodeling-directed interventions.

Future trials should incorporate clinically meaningful outcomes, including clinical worsening, hospitalization, transplantation, and mortality, together with measures of functional status, quality of life, and treatment burden.

Composite endpoints require particular attention. Their individual components should be prespecified, clinically justified, and transparently reported, with absolute event counts and component-specific estimates presented wherever possible. An overall composite treatment effect should not be interpreted as demonstrating equivalent benefit across all components.

Adequate follow-up is especially important when evaluating treatments intended to influence disease progression. Early termination, differential observation periods, and limited numbers of individual hard events can restrict conclusions regarding durability, mortality, and long-term safety.

Future research should also improve representation of patients with substantial cardiopulmonary comorbidities, different PAH etiologies, and characteristics underrepresented in pivotal trials. Dedicated studies may be needed to clarify benefit–risk relationships in low-risk populations and patients with greater bleeding or hematologic vulnerability.

These methodological improvements will be essential for distinguishing clinically meaningful treatment advances from promising but incompletely validated biological effects.

### 11.5. Translating Emerging Mechanisms into Clinical Benefit

Future therapeutic development may involve direct restoration of BMPR2 signaling, more selective growth-factor inhibition, immune and metabolic modulation, extracellular matrix targeting, mechanotransduction-directed approaches, and genetic or RNA-based interventions [3,9,10,12,32,33,34,35,36,37,38].

However, the development histories of imatinib and seralutinib demonstrate that mechanistic plausibility and favorable early-phase findings do not guarantee confirmatory clinical efficacy or acceptable safety.

Accordingly, future translational programs should incorporate evidence of target engagement, dose-dependent biological activity, and early safety characterization before proceeding to larger efficacy trials. Where biologically justified, prospective enrichment strategies may improve the identification of responsive patient populations.

The central research objective is not simply to expand the number of available molecular targets, but to establish which interventions produce meaningful clinical benefit, in which patients, and under what treatment conditions.

Successful translation will require integration of biological measurements with patient-important outcomes and rigorous comparison against contemporary standards of care.

## 12. Limitations

This review has several limitations that should be considered when interpreting its findings and clinical implications.

First, this study was conducted as a narrative review rather than a systematic review or meta-analysis. Although the literature synthesis prioritized randomized controlled trials, major clinical guidelines and consensus documents, prospective clinical investigations, and relevant mechanistic studies, study identification and selection were not conducted according to a prospectively registered systematic-review protocol. Complete database-specific search strategies, formal screening records, and study-selection counts were not preserved. Consequently, comprehensive study identification cannot be assured, and publication, language, and selection biases cannot be excluded.

Second, the available evidence varies substantially in methodological quality, patient populations, and clinical maturity. Sotatercept is supported by multiple randomized trials involving patients across different stages and severities of PAH [4,5,22,23,24,25]. In contrast, other remodeling-directed approaches are supported by heterogeneous combinations of preclinical investigations, early-phase clinical studies, inconclusive randomized trials, and preliminary phase III findings [3,9,10,12,28,29,30,31,32,33,34,35,36,37,38]. These differences preclude reliable indirect comparisons, formal ranking of therapeutic strategies, or conclusions regarding the relative superiority of distinct remodeling-directed mechanisms.

Third, heterogeneity across clinical trials limits cross-study interpretation. Differences in baseline risk, background therapy, disease duration, primary endpoints, composite-event definitions, and follow-up duration complicate comparisons of treatment effects. In particular, composite endpoint hazard ratios should not be interpreted as demonstrating equivalent reductions in mortality, transplantation, or hospitalization across different trials. Moreover, improvements in individual clinical endpoints do not necessarily establish comparable effects on long-term disease progression.

Fourth, some contemporary evidence remains incompletely reported. At the September 2026 literature cutoff, the phase III PROSERA trial of seralutinib had not been reported in a complete peer-reviewed efficacy publication. Although the available conference abstract described the trial design, numerical efficacy findings were obtained from a February 2026 sponsor announcement rather than a fully published clinical trial report [30,31]. The sponsor reported a placebo-adjusted improvement in six-minute walk distance of 13.3 m (*p* = 0.0320), which did not meet the prespecified primary significance threshold of α = 0.025 [31]. Consequently, the reported subgroup and secondary findings remain exploratory and require assessment in a complete peer-reviewed publication. Reliance on sponsor-reported topline findings limits independent evaluation of statistical analyses, outcome reporting, adverse events, and potential sources of bias.

Fifth, disease modification remains difficult to define and validate in PAH. Current studies predominantly evaluate clinical, functional, hemodynamic, biomarker, and right ventricular imaging outcomes rather than direct longitudinal changes in pulmonary vascular pathology. Although improvements in these measures may reflect clinically meaningful treatment benefits, they cannot independently establish structural reversal of pulmonary vascular remodeling or a sustained alteration in disease progression that persists independently of ongoing treatment [6,21]. Accordingly, the distinction between clinical improvement, biological pathway modulation, and demonstrated disease modification remains central to interpreting emerging therapies.

Sixth, the long-term effectiveness and safety of remodeling-directed treatment strategies remain incompletely characterized. Although extension studies provide additional information regarding sustained clinical observations and adverse events, their open-label designs, patient selection, and lack of concurrent randomized controls limit causal interpretation [23,40]. Furthermore, evidence remains insufficient to determine whether improvement following sotatercept initiation permits safe reduction or withdrawal of established background therapies, particularly parenteral prostacyclin treatment.

Finally, the evidence-calibrated interpretive framework presented in this review has not undergone prospective clinical validation. It is intended to distinguish guideline-supported interventions, individualized clinical decisions, and investigational therapeutic strategies according to the maturity and strength of available evidence. It should therefore be regarded as a conceptual framework for interpreting therapeutic developments rather than a validated clinical decision tool or an alternative to established professional guidelines.

This review reflects literature considered through September 2026. Its conclusions may require revision as complete peer-reviewed trial reports, longer-term follow-up data, real-world safety evidence, prospective studies of treatment sequencing and de-escalation, and updated professional recommendations become available.

## 13. Conclusions

Pulmonary arterial hypertension (PAH) management is evolving beyond conventional vasoactive therapy toward the integration of treatments targeting biological mechanisms implicated in pulmonary vascular remodeling. The endothelin, nitric oxide–cyclic guanosine monophosphate, and prostacyclin pathways remain the foundation of pharmacological management, while activin signaling inhibition has established a fourth clinically actionable therapeutic pathway [1,2,20].

Sotatercept represents a major therapeutic advance, providing randomized clinical evidence that modulation of remodeling-associated signaling can improve outcomes beyond established multidrug therapy. Its clinical development program has demonstrated benefits across hemodynamic, functional, biomarker, and clinical-outcome measures in different PAH populations [4,5,22,23,24,25]. However, these findings establish clinical efficacy rather than definitive structural reversal of pulmonary vascular remodeling in humans or treatment-independent disease modification.

Other mechanistically directed strategies, including growth-factor inhibition, BMPR2 restoration, immune and metabolic modulation, and extracellular matrix targeting, remain at varying stages of investigation. Their translation into routine practice requires evidence of clinically meaningful incremental benefit, acceptable long-term safety, and effectiveness alongside contemporary background therapy.

The central contribution of this review is an evidence-calibrated framework for interpreting the integration of mechanistically distinct therapies within risk-guided PAH management. By distinguishing guideline-supported interventions, individualized clinical considerations, and investigational approaches, this framework connects advances in pulmonary vascular biology with the strength of available clinical evidence without equating mechanistic plausibility with established therapeutic efficacy.

Future progress will depend not simply on expanding the number of therapeutic pathways, but on determining whether validated biological phenotypes and treatment-response biomarkers can improve therapeutic selection beyond conventional risk assessment. Until such strategies are prospectively validated, PAH management should remain grounded in established risk-guided principles, with remodeling-directed therapies incorporated according to demonstrated clinical benefit, safety, and individual patient characteristics.

## Figures and Tables

**Figure 1:**
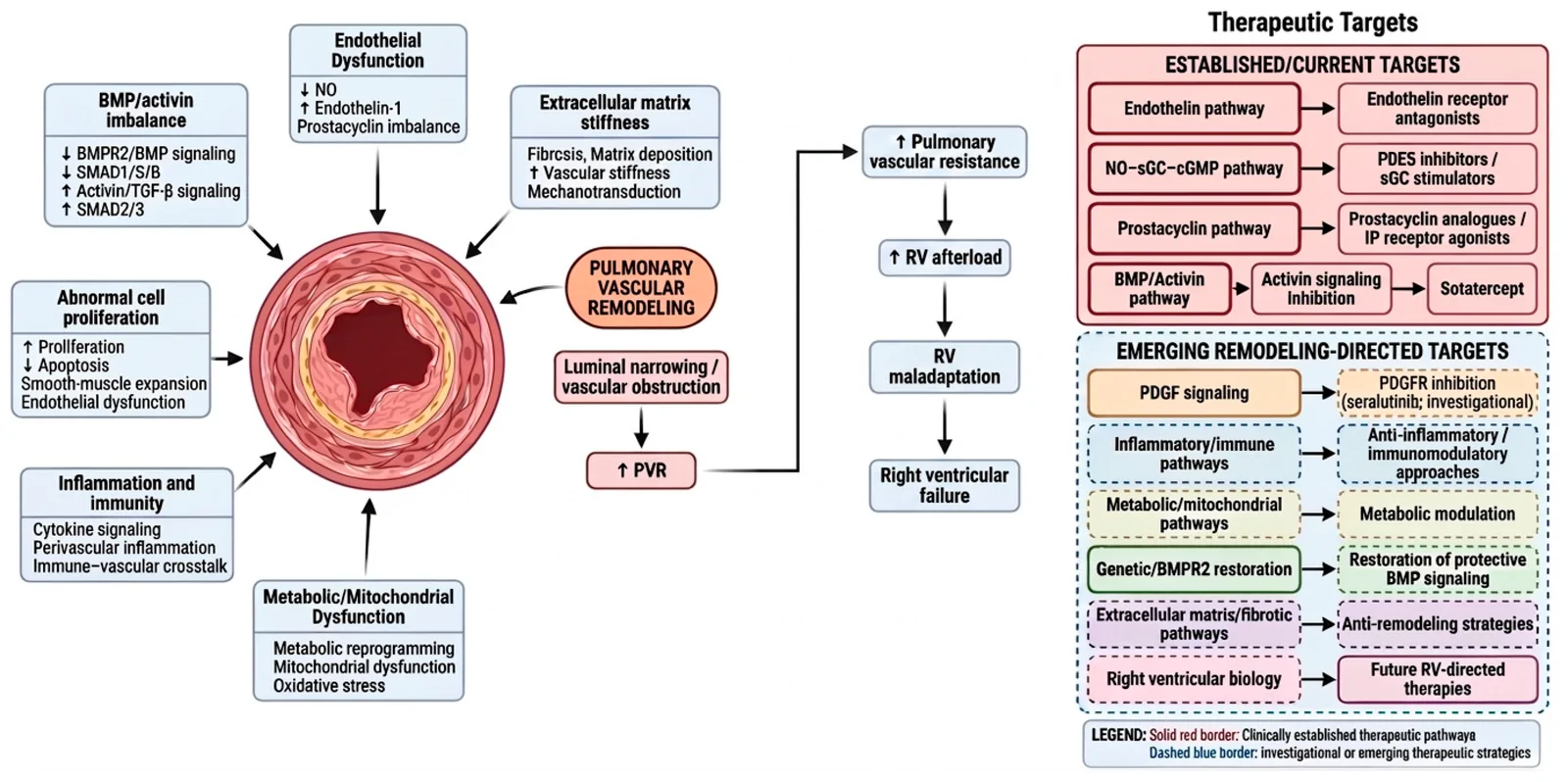
Pathobiological drivers of pulmonary arterial hypertension and established and emerging therapeutic targets. Endothelial dysfunction, dysregulated BMP/activin signaling, abnormal vascular cell proliferation, inflammation, metabolic dysfunction, and extracellular matrix stiffening contribute to pulmonary vascular remodeling, increased pulmonary vascular resistance, and progressive right ventricular dysfunction. Established therapeutic pathways include endothelin, nitric oxide–soluble guanylate cyclase–cyclic guanosine monophosphate, prostacyclin, and BMP/activin signaling. Emerging investigational strategies target PDGF signaling, immune and metabolic abnormalities, BMPR2 restoration, extracellular matrix remodeling, and right ventricular maladaptation. Solid red borders indicate established therapeutic pathways, whereas dashed blue borders identify investigational approaches with varying levels of clinical evidence. The figure is conceptual and does not imply equivalent efficacy or clinical readiness across targets.

**Figure 2:**
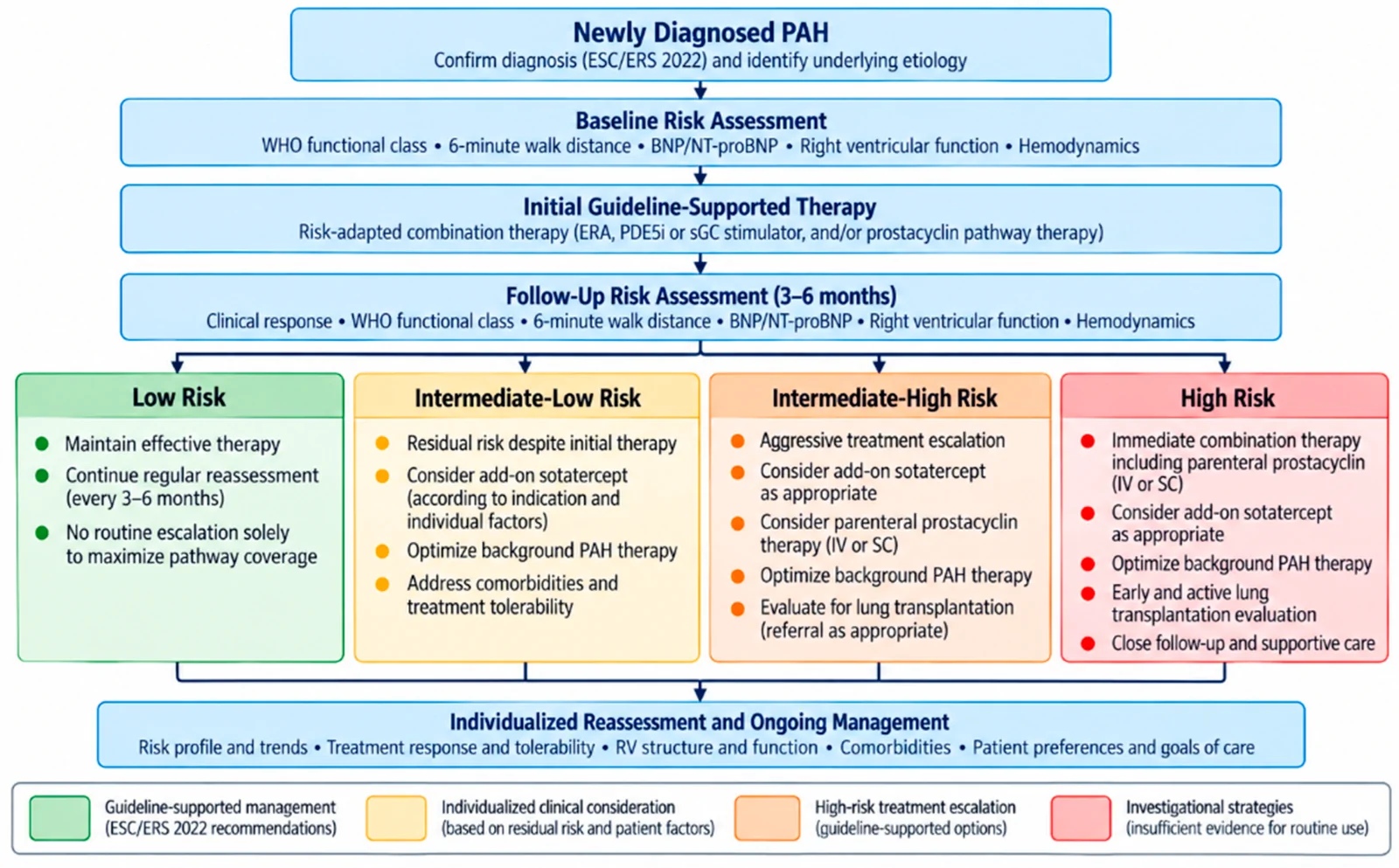
Integration of remodeling-directed therapy into risk-guided management of pulmonary arterial hypertension. Schematic representation of baseline risk assessment, initial guideline-supported treatment, follow-up risk stratification, and individualized therapeutic escalation in pulmonary arterial hypertension (PAH). Management is guided by clinical status, exercise capacity, biomarkers, right ventricular function, hemodynamics, comorbidities, and treatment tolerability. The framework distinguishes guideline-supported management (green), individualized clinical considerations (yellow), intensified guideline-supported treatment for higher-risk patients (orange), and investigational therapeutic strategies (red). Sotatercept may be incorporated according to residual risk and applicable recommendations, whereas parenteral prostacyclin therapy and timely lung transplantation evaluation remain important in higher-risk disease. The absence of a recommendation for sotatercept in low-risk patients reflects insufficient evidence rather than a recommendation against treatment. This figure represents an evidence-informed conceptual framework and has not been prospectively validated as a clinical treatment algorithm. Abbreviations: BNP, B-type natriuretic peptide; ERA, endothelin receptor antagonist; ESC/ERS, European Society of Cardiology/European Respiratory Society; IV, intravenous; NT-proBNP, N-terminal pro-B-type natriuretic peptide; PAH, pulmonary arterial hypertension; PDE5i, phosphodiesterase type 5 inhibitor; RV, right ventricle; SC, subcutaneous; sGC, soluble guanylate cyclase; WHO, World Health Organization.

**Table 1:** Evolution of the PAH treatment paradigm and pivotal supporting evidence.

Therapeutic Development	Representative Evidence	Principal Finding	Implications for Contemporary Management
Established vasoactive therapy	ERA, PDE5i/sGC stimulator, and prostacyclin clinical programs [1,2]	Demonstrated improvements in functional, hemodynamic, and clinical outcomes	Established three complementary therapeutic pathways
Initial dual oral combination	AMBITION [18]	Ambrisentan plus tadalafil reduced clinical-failure risk compared with pooled monotherapy	Supported early combination therapy in appropriately selected patients
Initial triple oral combination	TRITON [19]	Addition of selexipag to macitentan and tadalafil did not significantly improve the primary 26-week PVR endpoint	Demonstrated that increasing drug number does not necessarily produce incremental benefit
Risk-guided treatment escalation	ESC/ERS guidelines and World Symposium framework [1,2,15]	Repeated multiparametric assessment identifies residual risk and informs treatment decisions	Established longitudinal, goal-oriented management
Introduction of a fourth therapeutic pathway	World Symposium framework and sotatercept clinical program [2,4,5]	BMP/activin signaling became clinically actionable through sotatercept	Expanded therapeutic options beyond conventional vasoactive mechanisms
Risk-guided sotatercept incorporation	2026 ERS guideline update [20]	Recommends add-on sotatercept for patients at intermediate-low, intermediate-high, or high risk during follow-up	Integrates fourth-pathway therapy into evidence-based escalation while retaining conventional treatment foundations

Abbreviations: BMP, bone morphogenetic protein; ERA, endothelin receptor antagonist; ERS, European Respiratory Society; ESC, European Society of Cardiology; PAH, pulmonary arterial hypertension; PDE5i, phosphodiesterase type 5 inhibitor; PVR, pulmonary vascular resistance; sGC, soluble guanylate cyclase.

**Table 2:** Three-level framework for assessing therapeutic response and disease modification in pulmonary arterial hypertension.

Evidence Level	Principal Assessment Domains	Interpretation	Important Limitations
Level 1: Symptomatic or functional improvement	6MWD, WHO-FC, patient-reported outcomes, short-term biomarker changes	Demonstrates a favorable clinical response	May reflect reversible physiological effects or nonvascular factors
Level 2: Sustained disease control	Repeated risk assessment, PVR, cardiac output, NT-proBNP, RV imaging, adjudicated clinical-worsening events	Supports durable benefit during observed treatment	Does not directly demonstrate reversal of pulmonary vascular lesions
Level 3: Evidence supporting potential disease modification	Long-term clinical-event benefit, altered progression trajectory, biological target engagement, validated structural or molecular remodeling markers	Strengthens the inference that treatment influences underlying disease progression	No single currently validated measure proves disease modification; structural and biological evidence remains limited

Abbreviations: 6MWD, six-minute walk distance; NT-proBNP, N-terminal pro-B-type natriuretic peptide; PVR, pulmonary vascular resistance; RV, right ventricle; WHO-FC, World Health Organization functional class.

**Table 3:** Comparative clinical evidence for sotatercept in PAH.

Trial	Population and Background Therapy	Primary Endpoint and Follow-Up	Main Finding	Principal Limitations
PULSAR [22]	106 WHO-FC II–III patients receiving stable background therapy	PVR change at 24 weeks	Significant PVR reductions with both sotatercept doses	Small phase II study; surrogate primary endpoint
PULSAR extension [23]	97 participants entering open-label follow-up	Long-term clinical and hemodynamic assessment	Benefits maintained during continued treatment	Uncontrolled extension; cannot establish treatment-independent durability
SPECTRA [24]	Exploratory study with background PAH treatment	Exercise physiology and RV imaging	Favorable exercise-hemodynamic and RV measures	Small, nonrandomized study; limited causal inference
STELLAR [4]	323 WHO-FC II–III patients; substantial triple therapy and prostacyclin use	6MWD change at 24 weeks	Placebo-adjusted treatment difference of 40.8 m; favorable secondary outcomes	Limited primary follow-up; not designed to prove structural disease modification
ZENITH [5]	172 advanced high-risk patients on maximally tolerated treatment	Death, transplantation, or PAH hospitalization; event-driven	17.4% versus 54.7%; HR 0.24	Early termination; hospitalization contributes substantially to composite; limited component-specific precision
HYPERION [25]	320 recently diagnosed intermediate-/high-risk patients on dual or triple therapy	Clinical worsening; median follow-up 13.2 months	10.6% versus 36.9%; HR 0.24	Early termination; composite heavily influenced by exercise deterioration; no demonstrated mortality reduction

Abbreviations: 6MWD, six-minute walk distance; HR, hazard ratio; PAH, pulmonary arterial hypertension; PVR, pulmonary vascular resistance; RV, right ventricle; WHO-FC, World Health Organization functional class.

**Table 4:** Emerging therapeutic strategies targeting pulmonary vascular remodeling in PAH.

Therapeutic Strategy	Representative Intervention and Target	Evidence Maturity and Principal Findings	Major Limitations	Current Clinical Status
Systemic tyrosine kinase inhibition	Imatinib; PDGFR and related kinases [28]	Phase III randomized evidence of functional and hemodynamic improvement	Serious toxicity, treatment discontinuation, bleeding concerns	Investigational; not established for routine PAH treatment
Inhaled kinase inhibition	Seralutinib; PDGFRα/β, CSF1R, c-KIT [29,30,31]	Phase II PVR improvement; phase III PROSERA did not meet its prespecified primary statistical threshold	Unconfirmed phase III primary efficacy; subgroup signals exploratory; full dataset requires assessment	Investigational
Pharmacological BMP/BMPR2 restoration	Low-dose tacrolimus; BMPR2-associated signaling [32,34]	Preclinical activity and small phase IIa safety/feasibility study	Insufficient confirmatory efficacy evidence; safety and dosing uncertainties	Early clinical investigation
Gene-based BMPR2 restoration	BMPR2 gene delivery [33]	Preclinical improvement in experimental pulmonary hypertension	Delivery, durability, immunogenicity, genetic heterogeneity	Preclinical
Immune modulation	Rituximab; B-cell depletion in systemic sclerosis-associated PAH [35]	Randomized proof-of-concept trial with nonsignificant primary endpoint; exploratory later signals	Unconfirmed efficacy; uncertain responder phenotype	Investigational
Metabolic modulation	Metformin; metabolic and myocardial pathways [36]	Small uncontrolled mechanistic study with exploratory RV findings	No demonstrated functional efficacy; limited sample size	Early clinical investigation
Extracellular matrix and mechanotransduction targeting	Experimental YAP/TAZ, glutaminolysis, and matrix-directed approaches [12,38]	Predominantly preclinical evidence of effects on vascular proliferation and stiffness	No established human clinical efficacy	Preclinical
Epigenetic modulation	Experimental chromatin- and transcription-directed interventions [37]	Mechanistic and preclinical evidence	Target specificity, safety, and clinical translation	Preclinical
RV-directed therapy	Investigational approaches targeting myocardial metabolism, fibrosis, and adaptation [14,21]	Experimental and limited early clinical evidence	Difficulty distinguishing direct myocardial effects from pulmonary afterload reduction	Investigational

Abbreviations: BMP, bone morphogenetic protein; BMPR2, bone morphogenetic protein receptor type 2; CSF1R, colony-stimulating factor 1 receptor; PAH, pulmonary arterial hypertension; PDGFR, platelet-derived growth factor receptor; PVR, pulmonary vascular resistance; RV, right ventricle; YAP/TAZ, yes-associated protein/transcriptional coactivator with PDZ-binding motif.

**Table 5:** Practical safety monitoring and counseling considerations for sotatercept in PAH.

Safety Concern	Recommended Assessment	Practical Implication
Increased hemoglobin	Baseline hemoglobin; before each of the first five doses; continued monitoring if unstable; periodic assessment thereafter	Apply label-defined thresholds for treatment delay and reassess before restarting
Thrombocytopenia	Baseline and serial platelet counts, particularly during treatment initiation	Do not initiate below 50,000/mm^3^; delay treatment if counts fall below this threshold under U.S. labeling
Serious bleeding	Bleeding symptoms, history, platelet counts, and medication review	Do not administer during serious bleeding; evaluate and treat the underlying cause
Recurrent or unexplained gastrointestinal bleeding	Clinical evaluation, including consideration of gastrointestinal angiodysplasias	Consider endoscopic assessment where clinically appropriate
Epistaxis and other nonserious bleeding	Symptom assessment during follow-up	Evaluate severity, recurrence, and contributing factors
Telangiectasia	Clinical examination and long-term symptom review	Monitor progression and associated bleeding manifestations
Serious hypersensitivity	Assessment for anaphylaxis, angioedema, and other severe reactions	Discontinue sotatercept and initiate appropriate emergency treatment
Concomitant infused prostacyclin or antithrombotic treatment	Review background medications, platelet counts, and bleeding risk	Increase clinical vigilance and individualize safety assessment
Pregnancy and embryo-fetal toxicity	Pregnancy testing and reproductive counseling	Follow pregnancy-testing and contraception requirements in the approved label
Potential fertility impairment	Counseling before treatment initiation	Discuss possible effects on female and male fertility and uncertainty regarding reversibility
Breastfeeding	Counseling regarding potential infant exposure	Follow local labeling; U.S. guidance advises against breastfeeding during treatment and for four months afterward
Prolonged treatment interruption	Reassessment of hemoglobin and platelet counts before restarting	For interruptions exceeding nine weeks, follow the U.S. label’s lower-dose restart and escalation instructions
Long-term treatment exposure	Continued hematologic assessment, adverse-event surveillance, and clinical review	Monitor for delayed or cumulative adverse effects and reassess the benefit–risk balance

Abbreviations: PAH, pulmonary arterial hypertension. Note: This table summarizes practical considerations and does not replace approved prescribing information. Exact treatment-delay, dose-adjustment, restart, escalation, and discontinuation criteria must follow the locally applicable product label [39].

## Data Availability

No new data were created or analyzed in this study. Data sharing is not applicable to this article.

## Abbreviations

The following abbreviations are used in this manuscript:

Abbreviation	Definition
6MWD	6-min walk distance
BNP	B-type natriuretic peptide
BMP	Bone morphogenetic protein
BMPR2	Bone morphogenetic protein receptor type 2
cGMP	Cyclic guanosine monophosphate
CMR	Cardiac magnetic resonance
CSF1R	Colony-stimulating factor 1 receptor
ECM	Extracellular matrix
ERA	Endothelin receptor antagonist
ERS	European Respiratory Society
ESC	European Society of Cardiology
HR	Hazard ratio
NO	Nitric oxide
NT-proBNP	N-terminal pro-B-type natriuretic peptide
PAH	Pulmonary arterial hypertension
PDGF	Platelet-derived growth factor
PDE5i	Phosphodiesterase type 5 inhibitor
PDGFR	Platelet-derived growth factor receptor
PVR	Pulmonary vascular resistance
RHC	Right-heart catheterization
RV	Right ventricle
sGC	Soluble guanylate cyclase
SMAD	Suppressor of mothers against decapentaplegic
SSc-PAH	Systemic sclerosis-associated pulmonary arterial hypertension
TGF-β	Transforming growth factor beta
WHO	World Health Organization
WHO-FC	World Health Organization functional class
WSPH	World Symposium on Pulmonary Hypertension
